# Chimeric HP-PRRSV2 containing an ORF2-6 consensus sequence induces antibodies with broadly neutralizing activity and confers cross protection against virulent NADC30-like isolate

**DOI:** 10.1186/s13567-021-00944-8

**Published:** 2021-05-27

**Authors:** Nanhua Chen, Shubin Li, Yunfei Tian, Xinshuai Li, Shuai Li, Jixiang Li, Ming Qiu, Zhe Sun, Yanzhao Xiao, Xilin Yan, Hong Lin, Xiuling Yu, Kegong Tian, Shaobin Shang, Jianzhong Zhu

**Affiliations:** 1 College of Veterinary Medicine, Yangzhou University, Yangzhou, 225009 Jiangsu China; 2 Joint International Research Laboratory of Agriculture and Agri-Product Safety, Yangzhou, 225009 Jiangsu China; 3 Comparative Medicine Research Institute, Yangzhou University, Yangzhou, 225009 Jiangsu China; 4 Jiangsu Co-Innovation Center for Prevention and Control of Important Animal Infectious Diseases and Zoonoses, Yangzhou, 225009 China; 5 National Research Center for Veterinary Medicine, Luoyang, 471003 Henan China

**Keywords:** PRRSV, Infectious clone, ORF2-6 consensus sequence, Broadly neutralizing antibodies, Cross protection, Genetic engineered vaccine

## Abstract

Due to the substantial genetic diversity of porcine reproductive and respiratory syndrome virus (PRRSV), commercial PRRS vaccines fail to provide sufficient cross protection. Previous studies have confirmed the existence of PRRSV broadly neutralizing antibodies (bnAbs). However, bnAbs are rarely induced by either natural infection or vaccination. In this study, we designed and synthesized a consensus sequence of PRRSV2 ORF2-6 genes (ORF2-6-CON) encoding all envelope proteins based on 30 representative Chinese PRRSV isolates. The ORF2-6-CON sequence shared > 90% nucleotide identities to all four lineages of PRRSV2 isolates in China. A chimeric virus (rJS-ORF2-6-CON) containing the ORF2-6-CON was generated using the avirulent HP-PRRSV2 JSTZ1712-12 infectious clone as a backbone. The rJS-ORF2-6-CON has similar replication efficiency as the backbone virus in vitro. Furthermore, pig inoculation and challenge studies showed that rJS-ORF2-6-CON is not pathogenic to piglets and confers better cross protection against the virulent NADC30-like isolate than a commercial HP-PRRS modified live virus (MLV) vaccine. Noticeably, the rJS-ORF2-6-CON strain could induce bnAbs while the MLV strain only induced homologous nAbs. In addition, the lineages of VDJ repertoires potentially associated with distinct nAbs were also characterized. Overall, our results demonstrate that rJS-ORF2-6-CON is a promising candidate for the development of a PRRS genetic engineered vaccine conferring cross protection.

**Supplementary Information:**

The online version contains supplementary material available at 10.1186/s13567-021-00944-8.

## Introduction

Porcine reproductive and respiratory syndrome (PRRS) is an economically significant viral disease in the swine-producing countries of the world. The clinical symptoms are characterized by reproductive failure in sows and respiratory disease in young pigs [1]. The causative agent, PRRS virus (PRRSV), is one of the most rapidly evolving RNA viruses [2]. PRRSV can be divided into two species: PRRSV1 and PRRSV2, which share ~60% genomic similarity [3]. Furthermore, PRRSV1 has been classified into three subtypes, while PRRSV2 contains nine different lineages with genetic distances > 10% [4].

In China, even though PRRSV1 isolates were sporadically detected in recent years, PRRSV2 isolates were obviously predominant [5]. In 1995, PRRSV2 was first identified in Chinese swine herds [6]. In 2006, highly pathogenic PRRSV2 (HP-PRRSV2) variants first emerged in China, which were characterized by high fever (40–42 °C), high morbidity (50–100%) and high mortality (20–100%) in all ages of pigs [7]. Since 2013, NADC30-like PRRSV2 variants have become prevalent in China [8]. Since 2017, NADC34-like PRRSV2 isolates have also been detected [9]. To make matters worse, the coexistence of distinct PRRSV isolates within one pig herd or even within one pig has been identified in the field [5, 10].

Several commercial PRRS modified live virus (MLV) vaccines have been widely utilized in China, including RespPRRS MLV, CH-1R, R98, JXA1-R, HuN4-F112, TJM-F92 and GDr180. Generally, immunization with commercial PRRS MLV vaccines confers excellent homologous protection against closely related isolates but only limited cross protection against heterologous strains [11]. Due to the extraordinary ability of PRRSV to mutate and generate substantial genetic variations, the development of a broadly protective PRRS vaccine is particularly important to combat the continuously emerging PRRS outbreaks.

Neutralizing antibodies (nAbs) are a vital component of the immune armory against viral infection, which are induced against the viral outer coat proteins or envelope proteins [12]. The PRRSV virion surface contains at least seven envelope proteins. Open reading frames (ORF) 5 and 6 encode the major envelope proteins, GP5 and M, which form a disulfide-linked heterodimer. ORF 2, 3 and 4 encode the minor glycoproteins GP2a, GP3 and GP4 that form a noncovalent heterotrimer. Two small non-glycosylated proteins, E and 5a, are encoded by ORF2b and ORF5a, respectively [13]. Several studies have identified multiple neutralizing epitopes distributing among the major structural proteins (GP5 and M) and minor glycoproteins (GP2a, GP3 and GP4) [13–15]. In addition, the recognition of different neutralizing epitopes may induce homologous nAbs, heterologous nAbs or even broad nAbs (bnAbs) [13, 16, 17]. Similar results have been described in viruses such as human immunodeficiency virus (HIV) and influenza virus [18, 19]. PRRSV bnAbs have been found in sera from both naturally or experimentally infected pigs [12, 13]. However, PRRSV infection or vaccination generally induces delayed and ineffectual production of nAbs [12]. Specifically, bnAbs are only induced in about 1% of PRRSV infected pigs [17].

Multiple strategies have been employed to increase the breadth, potency and longevity of nAbs against rapidly evolving viruses, including epitope masking, sequential vaccination and centralized envelope antigens [19]. To overcome the extraordinary genetic diversity of PRRSV, several chimeric viruses were generated by molecular breeding of individual envelope protein (GP3, GP4, GP5, or M) from genetically divergent isolates, which could elicit heterologous cross-neutralizing antibodies [20–22]. However, the heterologous protection of these chimeric viruses in pigs was not assessed. A chimeric PRRSV containing multiple shuffled envelope genes could induce heterologous nAbs and confer partial cross protection against heterologous challenge [23]. According to the approach of centralized sequences [19], a PRRSV2 consensus full genome was designed and synthesized, which could confer broad levels of heterologous protection but the synthetic virus is highly virulent [11].

Here we hypothesized that a chimeric virus containing the consensus sequence encoding envelope proteins of PRRSV may induce broader nAbs. Therefore, a consensus sequence of ORF2-6 genes (ORF2-6-CON) encoding all envelope proteins of PRRSV was designed and synthesized. An infectious clone of avirulent HP-PRRSV2 isolate JSTZ1712-12 was first generated (named as rJSTZ1712-12) as we previously described [24, 25], and then used as the backbone for the construction of a chimeric virus containing ORF2-6-CON (designated as rJS-ORF2-6-CON). The chimeric virus is fully infectious in vitro and in vivo. More importantly, pig inoculation and challenge studies show that the rJS-ORF2-6-CON strain is avirulent, may induce bnAbs and confers satisfied cross protection against a virulent NADC30-like PRRSV isolate.

## Materials and methods

### PRRSV strains and cells

PRRSV strains used in this study were all stored in our laboratories including the following: PRRSV1: HLJB1 strain [26]; JXA1-like HP-PRRSV2: JSTZ1712-12 and JXA1-R strains [24]; CH-1a-like PRRSV2: SD1612-1 and CH-1R strains [27]; VR-2332-like PRRSV2: JSYC20-05-1 and R98 strains [27]; NADC30-like PRRSV2: SD17-36 and SD17-38 strains [28, 29]; NADC34-like PRRSV2: Anheal-1 strain (GenBank accession no. MH370474) was a courtesy from Dr Xizhao Chen at Beijing Anheal Laboratories Co. Ltd. Monkey kidney Marc-145 cells and baby hamster kidney 21 (BHK-21) cells were cultured in Dulbecco minimum essential median (DMEM) supplemented with 10% fetal bovine serum (FBS) and antibiotics. Pulmonary alveolar macrophages (PAM) were harvested by lung lavage from 6-week-old PRRSV-negative pigs [30]. PAM were maintained in Roswell Park Memorial Institute 1640 medium (RPMI-1640) supplement with 10% FBS and antibiotics.

### Design and synthesis of a PRRSV2 ORF2-6 consensus sequence

The approach so called “centralized envelope antigens” was adopted to overcome the extraordinary genetic diversity of PRRSV isolates [19]. To design a consensus sequence that contains the most common amino acids at each position of the envelope proteins, we collected 30 representative Chinese PRRSV isolates from GenBank. The ORF2-6 gene sequences encoding envelope proteins were aligned using ClustalX 2.0 [31]. The consensus ORF2-6 sequence (ORF2-6-CON) was generated using the DNAMAN 6.0 program. The ORF2-6-CON sequence was compared with the corresponding region in the JSTZ1712-12 strain (MK906026), frameshift mutations were manually revised to ensure the proper expression of all envelope proteins [11]. The ORF2-6-CON sequence was synthesized by the GENEWIZ Company (Suzhou, China).

### Construction and rescue of a chimeric HP-PRRSV2 with ORF2-6-CON

To generate an infectious clone carrying the ORF2-6-CON sequence, a full-genome cDNA clone of the avirulent HP-PRRSV2 isolate, JSTZ1712-12, was first constructed as we previously described [25]. Three overlapped fragments (F1, F2 and F3) spanning the full-length genome of JSTZ1712-12 were produced by PCR amplification using high fidelity PrimeSTAR MAX DNA Polymerase (TaKaRa, Shiga, Japan) with a panel of primers (Table 1). In the F3 fragment, a copy of the hepatitis D virus (HDV) ribozyme sequence was added immediately following the poly (A) tail via two rounds of PCR amplifications using JSTZ1712-12-AscI-F3/JSTZ1712-12-1R3 and JSTZ1712-12-AscI-F3/JSTZ1712-12-NotI-2R3 primer pairs. Four restriction enzyme sites (PacI, AflII, AscI and NotI) were used for assembling of the full-length JSTZ1712-12 clone (rJSTZ1712-12).

**Table 1:** **PCR primers used for the construction of PRRSV infectious clones.**

No	Primer	Sequence (5ʹ-3ʹ)^a^	Length (bp)
1	JSTZ1712-12-PacI-F1	AGCTCGTTAATTAATACATGACGTATAGGTGTTGGCT	37
2	JSTZ1712-12-AflII-R1	CATAGGTGCTTAAGTTCATTACCACCTGTAACGGAT	36
3	JSTZ1712-12-AflII-F2	ATCCGTTACAGGTGGTAATGAACTTAAGCACCTATG	36
4	JSTZ1712-12-AscI-R2	CCTTTCTGGCGCGCCCGAAAC	21
5	JSTZ1712-12-AscI-F3	GTTTCGGGCGCGCCAGAAAGG	21
6	JSTZ1712-12-1R3	***AGCGAGGAGGCTGGGACCAT****GCCGGCC*TTTTTTTTTTTTTTTTTTTTTAATTACGGCCGCATGGTTCT	68
7	JSTZ1712-12-NotI-2R3	ACAGGCGGCCGC*GTCCCATTCGCCATTACCGAGGGGACGGTCCCCTCGGAATGTTGCCCAGCCGGCGCC****AGCGAGGAGGCTGGGACCAT***^b^	89
8	PRRSV2-AscI-ORF2-CON-F3	TTTCGGGCGCGCCAGAAAGGGAAAATTTATAAAGCTAATGCCACCAGCATGAGGTTTCATTTTCCCCCGGGCCCT	75
9	PRRSV2-ORF6-CON-R	TTCTTTTTCTTTTGCTGCTTGCCGTTGTTATTTGGCATATTTGACAAGGTTTACCACTCCCTGT	64
10	PRRSV2-ORF6-CON-F2	ACAGGGAGTGGTAAACCTTGTCAAATATGCCAAATAACAACGGCAAGCAGCAAAAGAAAAAGAA	64

^a^The unique restriction enzyme sites used for cloning purposes are underlined.

^b^The hepatitis D virus ribozyme sequence is shown in italic and the overlapped region is highlighted in bold.

To construct the chimeric virus containing ORF2-6-CON, overlap extension PCR was performed to generate a recombined F3 fragment carrying the ORF2-6-CON. In details, the synthetic ORF2-6-CON sequence (in pUC57 vector) was amplified with the PRRSV2-AscI-ORF2-CON-F3/PRRSV2-ORF6-CON-R primer pairs to produce a fragment PRRSV2-AscI-ORF2-6-CON. In addition, the rJSTZ1712-12 clone was amplified with the PRRSV2-ORF6-CON-F2/JSTZ1712-12-NotI-2R3 primers to generate the JSTZ1712-12-ORF6-3ʹUTR-NotI fragment. Then an overlap PCR was performed with PRRSV2-AscI-ORF2-CON-F3/JSTZ1712-12-NotI-2R3 primers using the PRRSV2-AscI-ORF2-6-CON and JSTZ1712-12-ORF6-3ʹUTR-NotI fragments as templates to generate chimeric F3 containing ORF2-6-CON (ORF2-6-CON-F3). The recombined ORF2-6-CON-F3 was double-digested with AscI and NotI and then ligated into the rJSTZ1712-12 clone, which was linearized with the same restriction enzymes to generate the rJSTZ1712-12-ORF2-6-CON clone (abbreviated as rJS-ORF2-6-CON). The rJS-ORF2-6-CON genome sequence has been submitted to GenBank (MW460545).

The rJSTZ1712-12 and rJS-ORF2-6-CON clones obtained were transfected into BHK-21 cells using Lipofectamine 3000 Reagent (Invitrogen, USA) according to the manufacturer’s instructions. The culture supernatants collected at 48 h post-transfection (hpt) were serially passaged in Marc-145 cells. The recovery of the infectious viruses was confirmed by indirect immunofluorescence assay (IFA) [24]. PRRSV-specific murine mAb 15A1 (1:500 dilution) against the N protein was used as the primary antibody, while the Dylight 594 (Goat anti-mouse IgG, 1:1000, Invitrogen) was used as the secondary antibody. To study the growth kinetics of the viruses in vitro, Marc-145 cells were infected with 200 median tissue culture infectious doses (TCID_50_) of rJS-ORF2-6-CON, rJSTZ1712-12 or parental JSTZ1712-12 isolate, respectively. The multiple-step growth curves within 96 h post-infection (hpi) were determined by real-time RT-PCR assay [32]. The plaque morphology was examined in Marc-145 cells as previously described [33].

### Animal inoculation and challenge study

To evaluate the safety and immunogenicity of rJS-ORF2-6-CON, pig inoculation and challenge studies were performed. All animal experiments in this study were approved by the Animal Welfare and Ethics Committee of Yangzhou University with the Reference number of 202010001. Twenty 4-week-old PRRSV-free piglets were randomly divided into four groups (five piglets in each group). Piglets in the first group were intranasally and intramuscularly inoculated with 2 mL 10^5.0^ TCID_50_/mL rJS-ORF2-6-CON (5th passage). Piglets in the second group were injected with one dose of the commercial TJM-F92 vaccine (TECON, Xinjiang, China). Piglets in the other two groups were inoculated with RPMI-1640 to serve as the control. At 42 days post-inoculation (dpi), in addition to one RPMI-1640 inoculated group that was mock-infected again, the other three groups of pigs were challenged with 2 mL 10^5.0^ TCID_50_/mL of the virulent NADC30-like SD17-38 isolate [29].

Rectal temperature was recorded daily during the first 2 weeks both after inoculation and after challenge. The body weight was measured weekly and clinical signs were assessed daily. Serum samples were collected weekly for the analyses of virus load, antibody and IFN-γ levels. The dynamics of viremia were analyzed by PRRSV real-time RT-PCR assay [32]. PRRSV-specific antibodies in the sera were detected by HerdCheck^®^ PRRS×3 ELISA Kit (IDEXX, ME, USA). The threshold for seroconversion was set at a sample-to-positive (s/p) ratio of 0.4. In addition, IFN-γ in the sera were detected using the commercial Porcine IFN-gamma ELISA kit (ABSIN, Beijing, China) according to the manufacturer’s instructions. The weekly collected sera since 28 dpi were submitted to virus neutralization test as we previously described [13, 17]. The absence of a cytopathic effect (CPE) at a 1:8 dilution was considered positive for the presence of virus neutralizing activity. The pigs that survived until 14 days post-challenge (dpc) were euthanized and tissue samples including lungs, tonsils and lymph nodes were collected for histopathological and immunohistochemical examinations [7].

### Flow cytometric analysis of PRRSV-specific IFN-γ secreting cells

Swine periphery blood mononuclear cells (PBMC) were isolated from blood samples by density-gradient centrifugation as reported previously [34]. The isolated PBMC were plated in 96-well U bottom plates (2 × 10^6^ cells each well) and stimulated with or without HP-PRRSV2 MLV JXA1-R strain at a multiplicity of infection (MOI) of 0.01 for 20 h. Brefeldin A (10 μg/mL, Sigma, MO, USA) was added during the last 5 h of incubation. After incubation, the cells were harvested and stained for cell surface marker and intracellular cytokine as we previously reported [35]. Briefly, cells were stained with PerCP-Cy5.5-conjugated anti-pig CD3 (clone 8E6-8C8, BD Bioscience) for 30 min on ice. After washing, the cells were fixed with 4% paraformaldehyde and permeabilized with 0.2% saponin twice, and then incubated with Alexa Fluor 647-conjugated anti-pig IFN-γ (clone P2G10, BD Bioscience) or isotype matched control antibody for 20 min. Flow cytometry was performed with a FACS LSRFortessa (BD Biosciences, NJ, USA) and a minimal number of 300 000 cells was acquired. Data analysis was processed by FlowJo software (Tree Star Inc.).

### Characterization of distinct nAb VDJ repertoires

To characterize the swine VDJ repertoires associated with different PRRSV nAbs, in this study, we amplified the immunoglobulin heavy chain VDJ region from representative pigs producing distinct nAbs with a single pair of primers (pVDJ-FR1-F: 5ʹ-ATGGAGTTTCGGCTGAACT-3ʹ and pVDJ-FR4-R: 5ʹ-TGAGGACACGACGACTTCA-3ʹ) modified from our previous report [16]. The same strategies and criteria were used to analyze and select VDJ lineages potentially associated with different nAbs [16].

### Statistical analysis

The data of virus load, antibody level, rectal temperature, weight gain, IFN-γ concentration and percentage of IFN-γ secreting cells are shown as means ± standard deviations (SD). The differences between groups were determined by Mann–Whitney U test using Graphpad Prism version 6.07 [36]. A *p* value < 0.05 was considered statistically significant.

## Results

### ORF2-6-CON shares > 90% nucleotide identity to Chinese PRRSV2 isolates

**Figure 1:**
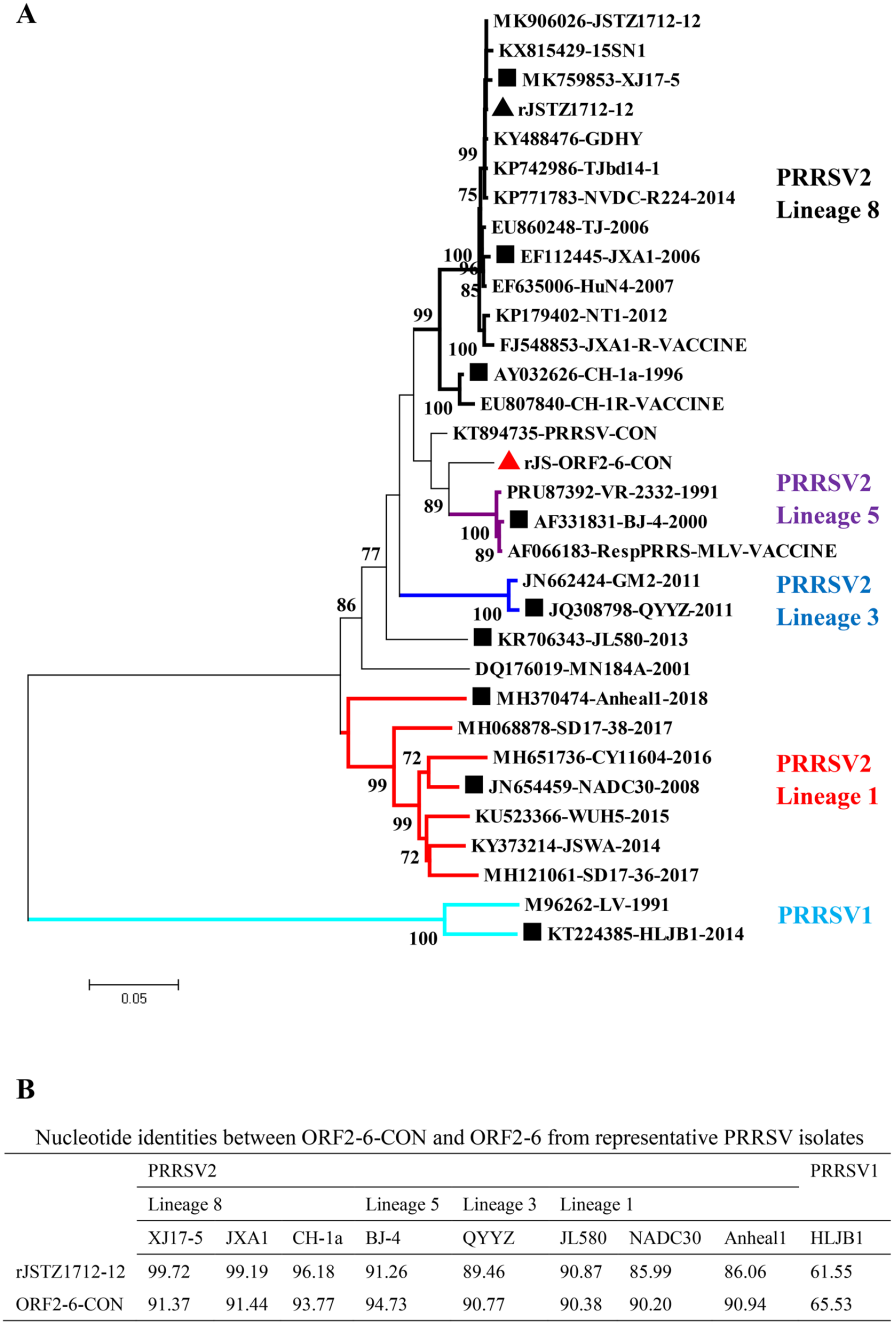
**Design of the consensus sequence of ORF2-6 genes (ORF2-6-CON)**. The ORF2-6-CON was designed according to 30 representative Chinese PRRSV isolates. A phylogenetic tree based on ORF2-6 genes was constructed (**A**). The ORF2-6-CON shared > 90% nucleotide identities to all four lineages of PRRSV2 isolates existing in China (**B**).

A set of 30 representative Chinese PRRSV isolates was obtained from GenBank. The phylogenetic tree was constructed based on ORF2-6 genes of mainly Chinese PRRSV2 and some representative PRRSV1 strains. There are four lineages (1, 3, 5 and 8) of PRRSV2 isolates co-existing in Chinese swine herds. The synthetic PRRSV2 ORF2-6-CON sequence was located at the center of the phylogenetic tree (Figure 1A), sharing > 90% nucleotide identities to all four lineages of PRRSV2 isolates and an increased nucleotide identity to PRRSV1 isolate (from 61.55 to 65.53%) (Figure 1B).

### The rJS-ORF2-6-CON is infectious in vitro

**Figure 2:**
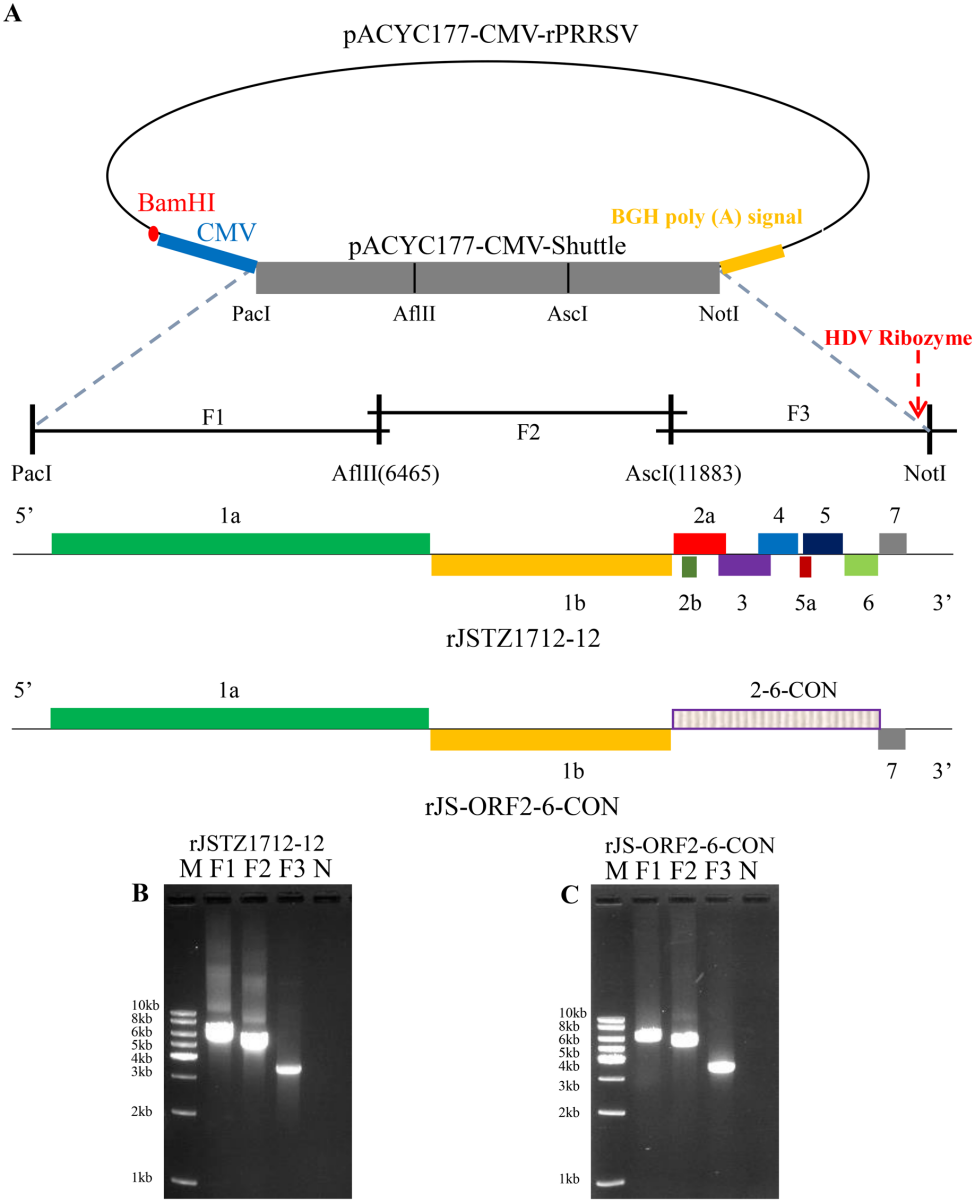
**Strategy to construct the full-length cDNA clones of avirulent HP-PRRSV2 JSTZ1712-12 isolate and a chimeric virus containing ORF2-6-CON**. The strategy was adopted from our previous study [25]. The pACYC177-CMV-Stuffer fragment including the unique restriction enzymes was shown in the upper part (**A**). The three overlapped fragments of the rJSTZ1712-12 (**B**) and rJS-ORF2-6-CON (**C**) genomes were produced by PCR amplification and are shown in the bottom part.

**Figure 3:**
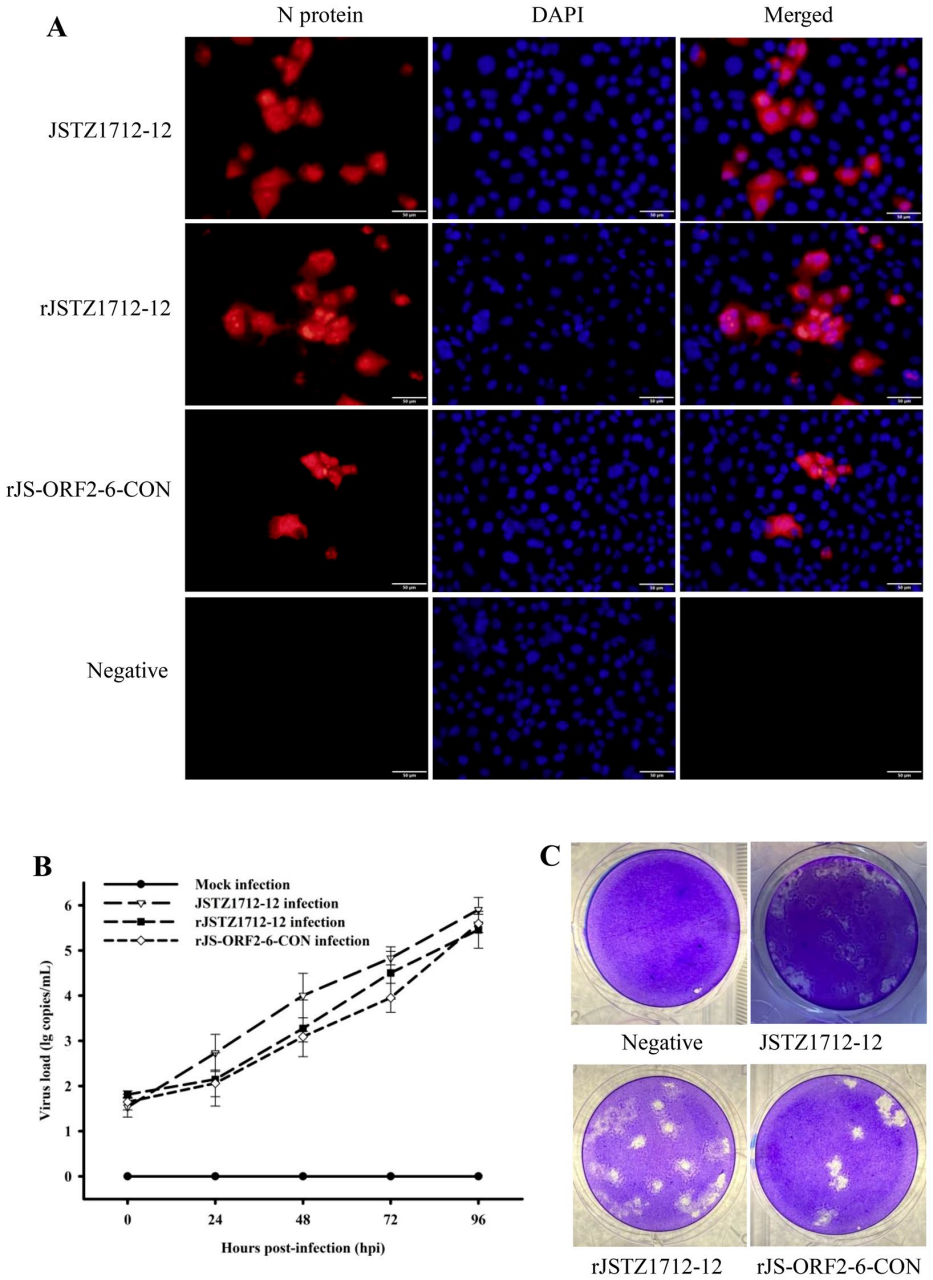
**Identification of the rescued rJSTZ1712-12 and rJS-ORF2-6-CON in vitro**. PRRSV-specific antigen was detected by the immunofluorescence assay (IFA) (**A**). The multiple-step growth curves in Marc-145 cells within 96 hpi were determined by real-time RT-PCR assay [32]. No significant difference was observed in the in vitro replication of the parental and cloned viruses (**B**). The JSTZ1712-12, rJSTZ1712-12 and rJS-ORF2-6-CON viruses have similar plaque morphology (**C**).

The rJSTZ1712-12 and rJS-ORF2-6-CON full-length cDNA clones were generated using the reverse-genetic system as we previously described (Figure 2) [25]. After five time passages in Marc-145 cells, typical PRRSV-specific cytopathic effects (CPE) could be observed at about 3–4 dpi. The presence of PRRSV was confirmed by IFA staining using cells infected for 48 h. PRRSV-specific antigen was detected in JSTZ1712-12, rJSTZ1712-12 and rJS-ORF2-6-CON infected cells but not in mock infected Marc-145 cells (Figure 3A). Multiple-step growth curves showed that the growth kinetics of parental JSTZ1712-12 isolate is similar (slightly higher but not significantly different, *p* > 0.05) to the cloned rJSTZ1712-12 and rJS-ORF2-6-CON viruses. The titers of all three viruses peaked at 96 hpi (Figure 3B). Furthermore, the rJS-ORF2-6-CON strain produced similar sizes of plaques as the backbone rJSTZ1712-12 virus and the parental JSTZ1712-12 isolate (Figure 3C). These results indicate that the cloned rJSTZ1712-12 and rJS-ORF2-6-CON viruses have similar in vitro growth characteristics to the parental JSTZ1712-12 isolate.

### The rJS-ORF2-6-CON is not pathogenic to piglets

**Figure 4:**
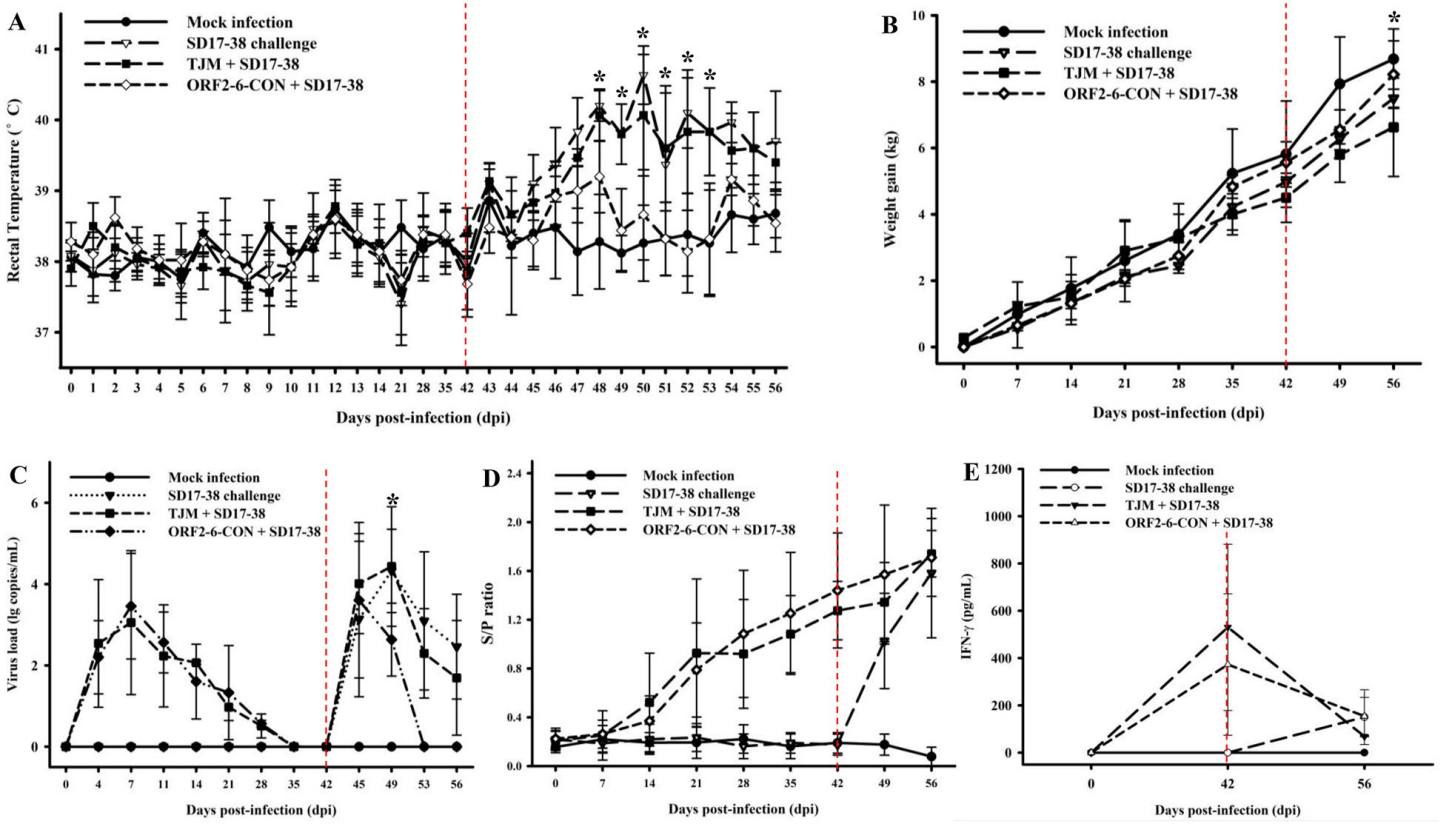
**Dynamics of rectal temperature, weight gain, virus load, antibody and IFN-γ levels during inoculation and challenge studies**. After the challenge of NADC30-like SD17-38 isolate, all positive control pigs and two of TJM-F92 vaccinated pigs showed high fever. However, the rectal temperature of rJS-ORF2-6-CON inoculated and mock-infected pigs did not reach 40 °C during the whole experiment (**A**). rJS-ORF2-6-CON inoculated pigs had significantly higher weight gain than TJM-F92 vaccinated pigs at 14 dpc (**B**). Dynamics of viremia were detected by real-time RT-PCR assay [32]. Compared with TJM-F92 vaccinated pigs, the virus load was significantly lower in rJS-ORF2-6-CON inoculated pigs since 7 dpc (**C**). PRRSV-specific antibody level was detected by IDEXX HerdCheck*PRRS×3 Antibody Detection ELISA Kit. The threshold for seroconversion was set at a sample-to-positive (s/p) ratio of 0.4. PRRSV-specific antibody could be detected in all PRRSV-infected pigs from 14 dpi to the end of the study (**D**). IFN-γ level was detected using a commercial Porcine IFN-gamma ELISA kit. No significant difference of IFN-γ amounts was detected in rJS-ORF2-6-CON inoculated and TJM-F92 vaccinated pigs at 42 dpi and 14 dpc (**E**). Each bar represents the average for all pigs in each group ± standard deviation (SD). The significant difference is marked with an asterisk.

Our previous study has shown that JSTZ1712-12 isolate is not pathogenic to piglets [24]. To characterize the pathogenicity of rJS-ORF2-6-CON in pigs, pig inoculation experiments were performed. Compared with the mock infected pigs, pigs inoculated with 5th passage of rJS-ORF2-6-CON did not result in any obvious clinical signs, or increase the rectal temperature, or affect the weight gain within 42 dpi (Figures 4A and B). Pigs inoculated with rJS-ORF2-6-CON virus had similar virus growth kinetics, similar levels of antibody and IFN-γ responses as in TJM-F92 vaccinated pigs (Figures 4C–E). These results indicate that the rJS-ORF2-6-CON virus is infectious in vivo, but is not pathogenic to piglets.

### rJS-ORF2-6-CON confers cross protection against a NADC30-like isolate

To evaluate the cross protection capacity of rJS-ORF2-6-CON, pigs in the rJS-ORF2-6-CON inoculated group, TJM-F92 vaccinated group and a control group were all challenged with the virulent NADC30-like SD17-38 isolate at 42 dpi. After challenge, the rectal temperatures of rJS-ORF2-6-CON inoculated pigs and mock-infected pigs were always lower than 40 °C, while the TJM-F92 vaccinated pigs and positive control pigs (only SD17-38 challenge) have significantly higher rectal temperatures (Figure 4A). Two out of the five TJM-F92 vaccinated pigs reached ≥ 40 °C (the highest of 41.1 °C), and all positive control pigs reached ≥ 40 °C (the highest was 41.3 °C). Clinical symptoms including dyspnea, anorexia and diarrhea were observed in the positive control pigs and two TJM-F92 vaccinated pigs (pigs 9 and 10), but not in mock-infected or rJS-ORF2-6-CON inoculated pigs. In addition, the weight gain of rJS-ORF2-6-CON inoculated pigs was not significantly different from the negative control group, but was significantly higher than TJM-F92 vaccinated pigs at 14 dpc (Figure 4B). SD17-38 virus was detected at 4 dpc in all challenged pigs, but the viremia was significantly lower in rJS-ORF2-6-CON inoculated pigs than in TJM-F92 vaccinated pigs since 7 dpc and could be eliminated within 11 dpc (Figure 4C).

**Figure 5:**
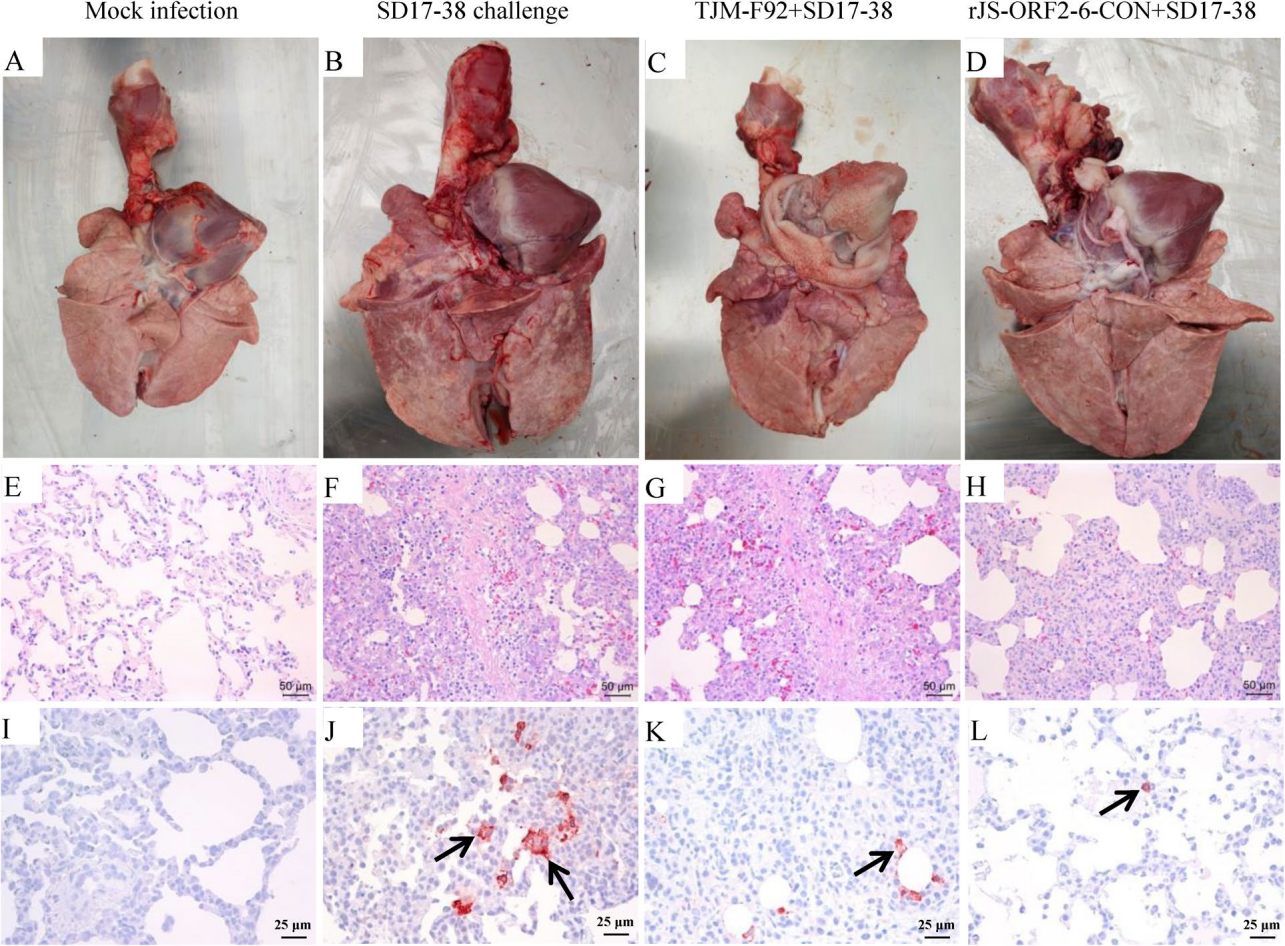
**Lung gross lesion, histopathological and immunohistochemical examinations**. Lung consolidation was observed in positive control and TJM-F92 vaccinated pigs (**B**, **C**) but not in mock-infected and rJS-ORF2-6-CON inoculated pigs (**A**, **D**). Interstitial pneumonia with infiltration of mononuclear cells could be observed in positive control and TJM-F92 vaccinated pigs (**F**, **G**) but not in mock-infected and rJS-ORF2-6-CON inoculated pigs (**E**, **H**). PRRSV-specific antigen could be observed in all PRRSV-infected pigs (marked by the black arrows) (**J**, **K**, **L**) but not in mock-infected pigs (**I**).

During the necropsy, lung consolidation was observed in positive control pigs and two TJM-F92 vaccinated pigs but not in mock-infected pigs or rJS-ORF2-6-CON inoculated pigs (Figures 5A–D). In addition, histopathological examination shows that interstitial pneumonia with infiltration of mononuclear cells was observed in the lungs from positive control pigs and TJM-F92 vaccinated pigs (Figures 5E–H). Furthermore, PRRSV antigen was detected in all challenged pigs but not in mock-infected pigs during the immunohistochemical examination (Figures 5I–L). The corresponding viruses in pigs during inoculation or challenge experiments were all confirmed by sequencing (data not shown). These results demonstrate that rJS-ORF2-6-CON confers better cross protection against virulent NADC30-like isolate than the TJM-F92 vaccine.

### The rJS-ORF2-6-CON induces similar frequency of PRRSV-specific IFN-γ secreting cells as the TJM-F92 vaccine

**Figure 6:**
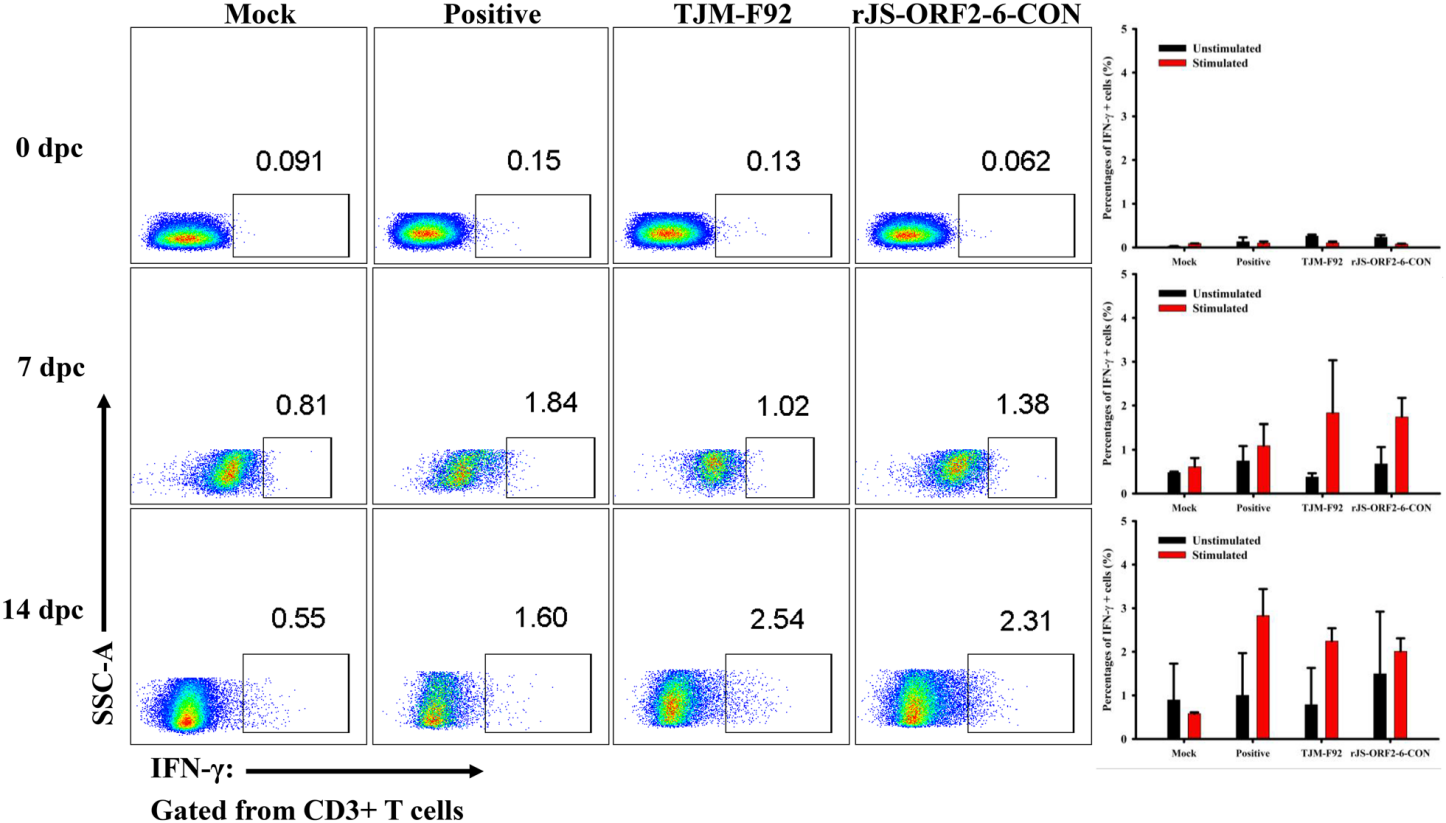
**Frequencies of PRRSV-specific IFN-γ secreting cells.** PBMC were isolated from each groups of pigs at 0 dpc (42 dpi), 7 dpc (49 dpi), 14 dpc (56 dpi) and stimulated with or without HP-PRRSV2 JXA1-R strain at 0.01 MOI for 20 h. The intracellular IFN-γ expression on total CD3+ T cells was further analyzed by flow cytometry. Representative dot-plots from each group were shown with frequencies of PRRSV-specific IFN-γ secreting cell population (left panel). The overall frequencies of PRRSV-specific IFN-γ secreting cells in each group were compared and are shown as bar graphs at the indicated time points (right panel). No significant difference was detected between rJS-ORF2-6-CON inoculated and TJM-F92 vaccinated pigs.

To evaluate the role of IFN-γ secreting cells in conferring cross protection, the frequencies of PRRSV-specific IFN-γ secreting cells in PBMC were tested by flow cytometric analyses. At 42 dpi (0 dpc), the frequencies of PRRSV-specific IFN-γ secreting cells was low and no significant difference was detected among all the groups. After challenge with the virulent SD17-38 isolate, the frequencies of PRRSV-specific IFN-γ secreting cells increased from 0 to 14 dpc. However, no difference was noticed between TJM-F92 vaccinated and rJS-ORF2-6-CON inoculated pigs (Figure 6). In addition, no significant difference was detected in IFN-γ concentrations of sera from TJM-F92 vaccinated and rJS-ORF2-6-CON inoculated pigs at 0 dpc and 14 dpc (Figure 4E).

### rJS-ORF2-6-CON induces antibodies with broadly neutralizing activity

To explore the role of nAbs in conferring cross protection, a virus neutralization test was performed for the 42 dpi (0 dpc) sera. No nAbs was found in any mock-infected pigs. One out of five pigs (pig 6) immunized with TJM-F92 vaccine induced homologous nAbs against HP-PRRSV2. Three out of five pigs (pigs 12, 13 and 14) inoculated with rJS-ORF2-6-CON virus induced heterologous nAbs against different lineages of PRRSV2 isolates (lineages 8, 5 and/or 1). Remarkably, pig 12 serum could neutralize not only distinct PRRSV2 isolates but also PRRSV1 isolate (Table 2). Therefore, pig 12 serum was described as possessing a broad neutralizing activity. Noticeably, the titers of nAbs in pigs 6, 12, 13, 14 increased but their neutralization activities did not change at 14 dpc (data not shown).

**Table 2:** **Virus neutralization properties of sera with distinct neutralization activities.**

PRRSV subgroups	Representative isolates	Mock inoculation	TJM-F92 inoculation	rJS-ORF2-6-CON inoculation
		Pig 3	Pig 6	Pig 12
JXA1-like HP-PRRSV2	JSTZ1712-12 (MK906026) (99/92)^a^	< 8^b^	64	32
	JXA1-R (MT163314) (99/91)	< 8	32	16
CH-1a-like PRRSV2	SD1612-1 (MN119304) (96/93)	< 8	< 8	32
	CH-1R (EU807840) (96/93)	< 8	< 8	16
VR-2332-like PRRSV2	JSYC20-05-1 (MT746146) (92/94)	< 8	< 8	16
	R98 (DQ355796) (92/94)	< 8	< 8	16
NADC30-like PRRSV2	SD17-36 (MH121061) (86/90)	< 8	< 8	8
	SD17-38 (MH068878) (85/90)	< 8	< 8	16
NADC34-like PRRSV2	Anheal-1 (MH370474) (86/90)	< 8	< 8	8
PRRSV1	HLJB1 (KT224385) (61/65)	< 8	< 8	8

^a^The numbers indicate the nucleotide identities between ORF2-6 sequences of TJM-F92 vaccine/chimeric virus (rJS-ORF2-6-CON) and ORF2-6 from representative PRRSV isolates, respectively.

^b^The neutralization titer shows an inverse of the highest serum dilution with virus neutralization activity. Titer < 8 indicates no detectable virus neutralization activity.

To further characterize VDJ repertoires associated with different PRRSV nAbs, the VDJ genes were amplified from two separate aliquots of each lymph node from four pigs secreting nAbs (pigs 6, 12, 13 and 14) and a mock infected pig (pig 3) as the control. A total of 271 swine VDJ sequences were obtained, including 55, 68, 56, 52 and 40 sequences from pig 3 that produced no nAbs, pig 6 for homologous nAbs, pig 12 for bnAbs, and pigs 13 and 14 for heterologous nAbs, respectively. All the swine VDJ sequences from this study have been submitted to GenBank (MW460272–MW460542). The alignment of VDJ sequences indicates that the divergence of swine VDJ genes was mainly due to high variation of the CDR (Additional file 1), which was consistent with previous reports [16, 37]. The diversification of the CDR was not only due to the high mutation rate but also to the change in length. The length of CDR1 was 5 aa, CDR2 was 7–9 aa, while CDR3 was 9–21 aa (Table 3).

**Table 3:** **VDJ sequences with identical or high similar CDR3 from four pigs.**

Pig	No	Name	CDR1	CDR2	CDR3
Pig 3	1	3-R3	SYAVS	YIGSSGR	ARGLAYGAIMDL
		3-F18	….	……	…………
	2	3-F13	STGII	EVTEDGGL	TLYLTYLDL
		3-F23	….	…….	………
	3	3-R29	SYEIS	GVDGDRWSG	AGCPLYSGCYIGQLGGVMDL
		3-R40	….	………	……………….
Pig 6	1	6-R10	DNAFS	AIASSDYDG	AIG-CYSYGASCYGSYYYAMDL
		6-F20	….	………	..QN………DQP--T…
	2	6-R27	GSYIN	TISSSGG	ATGLSMVLVAWGAMDL
		6-F28	….	……	……………
	3	6-F15	DYAFS	AIASSDYDG	ASAVAIAVTFGGRQQYYAMDL
		6-F17	….	………	…………………
Pig 12	1^a^	12-R3^b^	SYGVV	GIRIS-ISG	AGCAEYYFPYYYSVDL
		12-R10	..A.S	..DSGSY.	……W……M.
		12-R19	….	……S.	…N..W……I.
		12-R28	..A.S	..DSGSY.	……W……M.
		12-F3	.V…	A…….	………….M.
		12-F5	.V…	A…….	………….M.
Pig 13	1	13-R10	STYIN	FIGTGGA	ARGGCYIGYNCYDMHL
		13-F12	….	……	……V………
	2	13-R18	DYAFN	GTSKSDYDS	AVGGATIAVAIAVPNAMDL
		13-F7	….	………	………………
Pig 14	1	14-R14	SDPIG	RIFSGVS	AAYYEDTMHL
		14-F2	….	……	………
		14-F16	….	……	………
	2	14-R6	SYEIS	GIVSTGS	ARIAIPMVLAIPPYYTMHL
		14-R11	….	……	………………
		14-R17	….	……	………………
	3	14-R10	DTYIS	TISTADA	ARRDSGCANSYVD
		14-F19	….	……	…………
	4	14-R1	RTYIN	AISIGGV	ARDDFSDYCSASVCGMEL
		14-R12	.HD.	……	………………
	5	14-F1	SYPIG	DTSTSGG	ATGLMVLSSRTYGAMDL
		14-F5	….	……	…………….
	6	14-F11	RYEVT	GIDDGTG	AMSYTYGISYDYCGMDR
		14-F14	….	……	..G.S…………

^a^The shared and abundant sequences (≥ 3 sequences) are highlighted in red. The dot (.) means identical to the first aligned sequence. The short line (-) indicates a deletion. The column No. means the numbers of identical sequences from each pig.

^b^The names of VDJ sequences starting with R or F indicate that they are from two different aliquots. The following number denotes the obtained sequence number.

Capture–recapture analysis using two separate aliquots from the same pig lymph node was performed to refine the PRRSV activated B-cell clones [38]. Shared or identical sequences were identified in mock-infected pig 3 but were less abundant (< 3 sequences). Therefore, no sequence from the mock-infected pig met the criteria as we described previously [16]. In PRRSV-infected pigs, the most shared and abundant VDJ sequences were found in bnAbs pig 12 that has only one lineage including six highly similar CDR3, which met the shared and abundant criteria. Another lineage that met the criteria was identified in heterologous nAbs pig 14 with 3 identical CDR3. Even though shared sequences were also detected in pigs 6 and 13, they were not abundant with only two identical sequences. No lineage met the criteria found in pigs 6 and 13, which was probably due to not enough high depth coverage of sequencing.

## Discussion

Traditional PRRS vaccines cannot provide sufficient cross protection against high genetic divergent PRRSV isolates. Therefore, genetic engineering approaches have been widely applied to generate broadly protective vaccine candidates [11, 23]. In this study, we describe the generation of a chimeric HP-PRRSV2 virus containing a consensus sequence of PRRSV2 ORF2-6 genes, which could induce bnAbs and confer cross protection against a heterologous NADC30-like PRRSV2 isolate.

To overcome the big challenge caused by the substantial genetic diversity of PRRSV, multiple strategies have been employed in the last three decades. A multi-strain vaccine constructed based on five attenuated PRRSV strains was evaluated in pigs, which did not provide an improved cross protection compared with the single-strain vaccine and which might have a safety issue [39]. A chimeric PRRSV based on two field strains could confer better protection against both viruses but its efficiency against other heterologous strains was not tested [40]. DNA shuffling of individual envelope encoding genes (ORF3, ORF4, ORF5, ORF6) from multiple strains could generate chimeric viruses with improved cross-neutralizing antibodies [20–22]. The chimeric virus containing shuffled ORF3-6 genes conferred an enhanced cross protection in pigs against NADC20 and RFLP 1-7-4 strains [41]. A consensus PRRSV genome was designed using the strategy of centralized sequences based on 59 PRRSV2 genomes. The generated PRRSV-CON strain is highly virulent but it could confer significantly broader levels of heterologous protection than the wild-type strain [11]. In this study, we synthesized an ORF2-6 consensus sequence of PRRSV2 encoding all envelope proteins. The infectious clone of avirulent HP-PRRSV2 JSTZ1712-12 strain was used as the backbone to generate a chimeric virus containing the ORF2-6-CON. The rJS-ORF2-6-CON strain was not pathogenic in pigs, it might induce bnAbs and could confer cross protection against the virulent NADC30-like SD17-38 isolate. Our results indicate that rJS-ORF2-6-CON may be a promising candidate for the development of a broadly protective PRRS vaccine.

A huge amount of PRRSV strains has been isolated in Chinese swine herds. PRRSV2 is predominant since its emergence in 1995 and causes several outbreaks in the last two decades [7, 8, 42, 43]. The HP-PRRS pandemic caused by HP-PRRSV2 has seriously prejudiced the development of the Chinese swine industry since 2006 [7]. In 2013, the emergence of NADC30-like PRRSV in China caused enormous losses due to the limited cross protection of commercial PRRS vaccines against NADC30-like isolates [8, 44–46]. Currently, the prevalent Chinese PRRSV2 isolates can be divided into four lineages (lineages 1, 3, 5 and 8) [47]. In addition, PRRSV1 isolates have been detected in more than ten provinces and have evolved into at least four subgroups in China [26, 30, 48]. The co-existence of highly divergent PRRSV isolates makes the control of PRRS more complicated and difficult in China. Here we designed an ORF2-6 consensus sequence based on 30 Chinese PRRSV isolates representing different species and lineages. Intriguingly, the chimeric virus could induce bnAbs against both PRRSV1 and different lineages of PRRSV2 isolates.

Previous studies demonstrated that nAbs plays a critical role in PRRSV protective immunity [49, 50]. Passive transfer of PRRSV nAbs alone could fully prevent the transplacental infection with PRRSV and provide sterilizing immunity in vivo [50]. Multiple neutralizing epitopes have been identified among PRRSV major and minor envelope proteins. Several studies showed that minor glycoproteins GP2, GP3 and GP4 of PRRSV1 strains possess neutralizing epitopes, while GP5 and M heterodimers of PRRSV2 strains are the major target for viral neutralization [15, 51]. PRRSV infection might induce homologous nAbs, heterologous nAbs and bnAbs [13, 17]. Remarkably, pigs exposed to circulating field PRRSV strains could induce bnAbs against both PRRSV1 and PRRSV2 [12]. Our previous studies also showed that ~1% of experimentally infected pigs could produce bnAbs [13, 16]. However, what strategy should be used to induce bnAbs remains unclear. In this study, we show that the rJS-ORF2-6-CON strain could induce heterologous nAbs or even bnAbs. The results demonstrate that the designation of a consensus sequence encoding all PRRSV envelope proteins could be an alternative strategy to inducing broader cross-neutralizing antibodies. In addition, this chimeric virus may be a useful tool to identify the exact epitope/residue involved in inducing distinct nAbs.

Pigs that produce homologous nAbs, heterologous nAbs and bnAbs provide an ideal opportunity to explore swine B cell repertoires associated with different nAb responses. To elicit immune response to unlimited numbers of foreign antigens, the immune system must recognize countless numbers of antigens. The unlimited numbers of unique antigen receptors are achieved by creating variation in the antigen-recognition regions [52]. The mechanisms mainly involve mixing and matching variable (V), diversity (D), and joining (J) gene segments in a process called V(D)J recombination. Antigen-binding specificity of an antibody is primarily determined by its heavy chain variable regions [53]. Only a few genes are involved in antibody production in pigs. Swine utilize seven major V_H_ genes (V_H_A, V_H_B, V_H_C, V_H_E, V_H_F, V_H_Y, V_H_Z), two D_H_ segments and a single J_H_ gene to account for nearly its entire (>90%) VDJ pre-immune repertoire [54]. Furthermore, exposure to environmental antigen does not change the V_H_ genes that comprise the pre-immune repertoire. The same V_H_ genes comprise the adaptive repertoire but ~90% of them are somatically mutated [54]. These unique features provide an opportunity to analyze the porcine antibody repertoire by detecting the entire VDJ repertoire using a single primer set [16]. In this study, we determined shared and abundant VDJ genes from pigs secreting bnAbs and heterologous nAbs, these shared and abundant sequences were likely expressed by B cells activated by PRRSV infection. B cells from different lymphoid tissues of a PRRSV-infected pig sampled at the same time displayed a similar pattern, indicating the widespread dissemination of the same B cell clones [55–57]. Therefore, B cell repertoires of the lymph nodes could represent the entire B cell repertoires of PRRSV-infected pigs, suggesting that the shared and abundant sequences in these pigs were potentially correlated with the different nAb responses. However, further studies, such as the construction of single-domain antibodies, need to be executed to determine whether the shared and abundant VDJ lineages identified in this study are PRRSV-specific or distinct nAbs-associated.

Cross protection is extremely important due to the coexistence of distinct PRRSV isolates. However, by which mechanisms to confer protection by PRRSV are poorly understood [58]. PRRSV-specific IFN-γ producing cells have been suggested to be correlated with vaccine-induced protection [59]. However, the degrees of correlation between the frequencies of PRRSV-specific IFN-γ secreting cells and the levels of protection are highly variable [60]. In this study, rJS-ORF2-6-CON could confer better cross protection than the TJM-F92 vaccine, but no significant difference was detected in IFN-γ secreting cells and IFN-γ production by these two groups of pigs. In contrast, rJS-ORF2-6-CON could induce heterologous nAbs or even bnAbs while TJM-F92 only induced homologous nAbs. These results indicate that nAbs play a critical role in conferring cross protection. Passive transfer studies provided direct evidence that nAbs alone can protect pigs against PRRSV infection [49, 50]. It would be interesting to evaluate whether heterologous nAbs or bnAbs alone can provide cross protection or whether they need assistance from other immune factors in the future.

Even though protection efficiency is a vital criterion for breeding next generation PRRS vaccines, safety is another major concern that is at least as important as protection efficiency. Persistent MLV infection in vaccinated pigs and transmission of vaccine strains to naïve pigs have been confirmed [27, 51, 61]. In addition, MLV-derived field isolates and recombinants from MLV and wild-type strains have been isolated [26, 62]. Moreover, some MLV-like isolates (NT1, JX2014T2 and XJ17-5) are highly virulent viruses determined by animal challenge studies [24, 63, 64]. Therefore, more attention should be paid to PRRS vaccine safety. Even though the preliminary results from this study show that rJS-ORF2-6-CON is not pathogenic to piglets, systematic safety tests must be performed to decide whether it can be used as a vaccine in pigs.

In conclusion, an ORF2-6 consensus sequence was computationally designed based on a large amount of representative Chinese PRRSV isolates in this study. The chimeric virus containing this consensus sequence is not pathogenic to piglets, may induce bnAbs and confers better cross protection against heterologous NADC30-like isolate than a commercial MLV vaccine. The chimeric virus may serve as a promising candidate for developing broadly protective PRRS vaccine. In addition, this chimeric virus can be used to investigate the mechanisms involved in inducing bnAbs or conferring cross protection.

## Supplementary Information

**Additional file 1. Alignment of 271 porcine VDJ amino acid sequences from this study.** Overall, the variation of swine VDJ genes is concentrated in the CDR (A). The detailed alignment of 271 VDJ (B). The identical sequences from each pig are highlighted in red.

## Data Availability

All the data generated or analyzed during the study are included in this article. The datasets used in the present research project are available from the corresponding authors upon reasonable request.

## Abbreviations

PRRSV: Porcine reproductive and respiratory syndrome virus
HP-PRRSV2: Highly pathogenic porcine reproductive and respiratory syndrome virus 2
HIV: Human immunodeficiency virus
nAbs: Neutralizing antibodies
bnAbs: Broadly neutralizing antibodies
MLV: Modified live virus
ORF: Open reading frame
CON: Consensus sequence
GP: Glycoprotein
VDJ: Variable diversity joining
CDR: Complementary-determining region
NADC: National animal disease center
DMEM: Dulbecco’s minimum essential median
FBS: Fetal bovine serum
PAMs: Pulmonary alveolar macrophages
CPE: Cytopathic effect
BHK-21: Baby hamster kidney 21
Marc-145: An African green monkey kidney cell line
hpi: Hours post-infection
hpt: Hours post-transfection
dpi: Days post-infection
dpc: Days post-challenge
IFA: Indirect immunofluorescence assay
TCID_50_: Median tissue culture infectious doses
MOI: Multiplicity of infection
PBMCs: Periphery blood mononuclear cells
IFN: Interferon
ELISA: Enzyme-linked immunosorbent assay
RPMI: Roswell Park Memorial Institute
HDV: Hepatitis D virus
SD: Standard deviations
