# Supplementary material for: Chimeric HP-PRRSV2 containing an ORF2-6 consensus sequence induces antibodies with broadly neutralizing activity and confers cross protection against virulent NADC30-like isolate
Source: Vet Res. 2021 May 27;52:74. doi: 10.1186/s13567-021-00944-8 (PMC8161975; doi:10.1186/s13567-021-00944-8)
Supplement: Supplementary file 1 — Additional file 1. Alignment of 271 porcine VDJ amino acid sequences from this study. Overall, the variation of swine VDJ genes is concentrated in the CDR (A). The detailed alignment of 271 VDJ (B). The identical sequences from each pig are highlighted in red. [file 13567_2021_944_MOESM1_ESM.pdf]

A

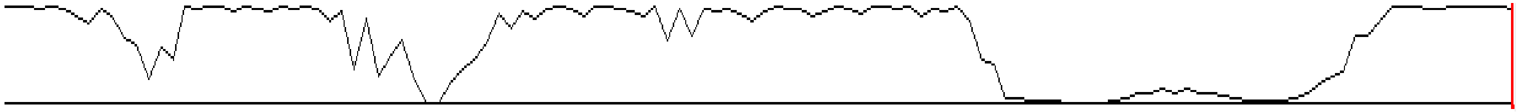

B

```
                   CDR1           CDR2                                                CDR3
Pig3-VDJ-R5    SCVGSGFDFSDNAFSWVRQAPGKGLEWVAAIGTSD--YDGRTYYADSVKGRFTISRDDSQNTVYLQMNSLRTEDTARYYCAKGGMV----AMTVAGFVMN--------YYNLHLWGPGVEVVVSS [125]
PIG14-VDJ-F18  ...........T.........R.......LIG.--...S.............S..........D.............IR.-----------------------.LQMN........... [125]
Pig6-VDJ-R10   .............................AS..--...S.............S.........................I.-------CYSYGASCYGSYY------.AMD........... [125]
Pig6-VDJ-F20   .............................AS..--...S.............S.........................IQN------CYSYGASCYDQP--------TMD........... [125]
Pig13-VDJ-F1   ........L..-T................AS..--...S.............S....M....................I.PLG----CYSYRASCY----------AMD........... [125]
Pig13-VDJ-F19  ..........S..................AN..--...SI.V...M.......S.NV.......................I.N------CY.YGASCYALGVG----..AMD........... [125]
Pig12-VDJ-R27  .............................SSR.--...S..N...........S.N........................IP.-------DSYG-GLY----------AMD........... [125]
Pig6-VDJ-F35   .............................AS..--...S.............S.N........................IDRNY--------GTTAWW---------DA.D........... [125]
Pig13-VDJ-R19  .............................VIG.--...S.............S.N........................TI.---------------------------.DV........... [125]
Pig12-VDJ-R17  .............................AS..--..SS.............S.N........................I.R------WVAI.VTPR-----------MD........... [125]
PIG14-VDJ-F10  .............................AS..--...S.............S..........................I.KRNY--I.VAI.VTPIPTSVYRYAH..AMD........... [125]
Pig13-VDJ-R13  ..............N..............A...--...S.............S..........................IW.I----------------------A.TMD........... [125]
Pig13-VDJ-F28  .............................VS..--...S.............S.K........................I.EIN----MVL..MMTTT--------M.VMD........... [125]
Pig13-VDJ-F8   ....P.I.....D................A...--...N...S..........N.N........................MKVVT-----AA..IA.HH----------AME....D...L... [125]
Pig13-VDJ-F30  ..........V..T...............AG.G--.A...............S.Y.........................R.-------QAA.IAFTG----------MD........... [125]
Pig13-VDJ-R18  ..........Y..N.......N.....GTSK..--..SD.S............S....M....................V..AT-----IA..IA.P.----------AMD........... [125]
Pig13-VDJ-F7   ..........Y..N.......N.....GTSK..--..SD.S............S....M....................V..AT-----IA..IA.P.----------AMD........... [125]
Pig6-VDJ-R26   ..........Y..................AS..--.N.S.............S....M...................VYS.--------CYS..Y-------WAF..AMD........... [125]
Pig13-VDJ-F3   ..........Y..................ASG.--..NS.............S....M...................VCS---------CYS.-----------YGDAMN........... [125]
Pig6-VDJ-F15   ..........Y..................AS..--...S.............S....M....................SAV--------AI.VTFGG-----RQQ..AMD........... [125]
Pig6-VDJ-F17   ..........Y..................AS..--...S.............S....M....................SAV--------AI.VTFGG-----RQQ..AMD........... [125]
Pig13-VDJ-F21  .....DL....Y.................AS..--H.....S..........S....M....................IAV--------AN.NT-------------GMN........... [125]
Pig12-VDJ-F15  ..........Y.........R........H...--...S.............S....M....................IAE--------Q-------------DLG.IMD........... [125]
Pig13-VDJ-R6   ..........Y..................AS..--...S.............S....M....................IR-----------------------LLLFMD........... [125]
Pig13-VDJ-R26  .......T...Y.................AS..--...S.............S....M....................Q..--------SYGAS---------SW.HAMD........... [125]
Pig6-VDJ-F38   ..........Y................T.TS.A--...S.............S....MM...................MAT--------I.V.---------IGY---MD........... [125]
Pig12-VDJ-F11  ..........Y................T.AN..--...S.............S.N..M....................M.R--------LLRS---------WPY..SMD........... [125]
Pig12-VDJ-R18  ..........Y...................ST.--..................S....IM...................FI--------A..IAAG------WSSS.AMD........... [125]
Pig13-VDJ-R37  .......T...YT................VR..--...S........G....S......L..................VMSI--------A.RQSG------------PMD........... [125]
```

```
Pig12-VDJ-R1   .......N.R.R..T................VSG.--WT.S...........L.S......S.................G.P-------NLYPLLSRP---------RDMD...........  [125]
Pig12-VDJ-R14  ......IT..SY.V..................K..--...S.............S.N......................I.----------FDSGYPY--------..VMDF..........  [125]
Pig12-VDJ-F12  .......T..SYEIN................AS..--...S.............S......H.................I-----------.PRGYPP--------..AMD...........  [125]
Pig3-VDJ-R29   .......T..SYEI.............L.GVDGDRW--S.T.N..............N...A.................GCP----LYSGCYI.Q----------LGGVMD...........  [125]
Pig3-VDJ-R40   .......T..SYEI.............L.GVDGDRW--S.T.N..............N...A.................GCP----LYSGCYI.Q----------LGGVMD...........  [125]
Pig12-VDJ-F28  .......T..SYEI----------------------------------------K.N...A..................NY----DYIGCLT-------------G.EMN...........  [125]
Pig3-VDJ-F14   .......T..NYEIT............L.T..CAGY--R.I.D....R........ND..A.................T.------QSGCYS.Y----------MGDGMD...........  [125]
Pig6-VDJ-R16   ......YT..SY.IG............LST.N..G----.K...............N...A.................TA------YSGCYS.Y----------V-----E..........  [125]
Pig3-VDJ-F6    .......T..TYFIG............L.S.RPD----S.D.......R........N...A........A........ERQ--------IYY----------------GMN...........  [125]
Pig6-VDJ-R6    .......T..SYTV............GL.L.RS-----S.T.............K.N...A...............F..RV--------GYYSNA----------FAEGMD...........  [125]
Pig12-VDJ-R11  .......T..SYEIN............LGD.V..----S.S.LH..........S.N...A.................RSVD--IYDISCW-------------FFCKMD...........  [125]
Pig13-VDJ-R36  .......T..SYEI.............L...R..----.SS...........M.S.K...A.................R.LY--SYY.SCFG.A----------CS.AMD...........  [125]
Pig6-VDJ-R12   .......T..STYIN........A...L.G.YSV----GTS...T...E.....K.N...A.................S.--SYRYG.SCYFV.------------CAMD...........  [125]
PIG14-VDJ-R29  .......T..SYEI.............L.G.NGV----GRN...I.........K.N...A.................G.L-.YNYRSGE----------------WAMD...........  [125]
Pig12-VDJ-F31  .......T..SYSM.............LTG.YS.----ASS.............S.N...A.................RT-SCYSYG.RCYDDPL--------RY..AMD...........  [125]
PIG14-VDJ-R31  .......T..NY.V.............L.C.SS.----GSS...............N...A......P...........R--GCYSYG.ACYP----------------MD...........  [125]
Pig12-VDJ-F18  .......T..TYSM.............L.C.SS.----GSS...............N...A.............H.F..R-----GYGISCY.DT--------------MY...........  [125]
Pig13-VDJ-F14  .......T..SYEI..........P..L.G.YS.----GSS...............N...A.................RPMGCYTYGVSCYYYA--------------MD...........  [125]
Pig13-VDJ-F15  .......T..SYEI.............L.G.YSG----GSS.............S.N...A.................R--GCLGYGVSCYSDS--------------MD...........  [125]
Pig13-VDJ-R27  .......T..SYEIV............M.I.N..----G.S...V.........K.....S.................R.-SCYSYG.SCYEEG-----------GFAMD...........  [125]
Pig13-VDJ-R29  .......T..SYEI.............L.T....----GSS.............K.N...A.....G............R--ICYSYG.SCYPK------------..AMD...........  [125]
Pig3-VDJ-R21   .......T..SYEI.............L.G.YS.G----SS...............K...A.................R.R-------DYSGCCGP-VRG-------AMD....S.......  [125]
Pig6-VDJ-R38   .......T..SYPIN............L.G.YS.G----SS.............S.N...A....RR.......H....R.R-------NWSGW.G--YYG-------AMD...........  [125]
PIG14-VDJ-R22  .......T..SYPIG............L.G.YP.G----ST.............S.K...A................TR.----------SS.CRC-ADC-------TM............  [125]
Pig6-VDJ-R41   .......T..SYEI.............L.G.YS.G----SS...............N...A........I..M......T.R-------LL..MMTTGPYY-------PMN...........  [125]
Pig13-VDJ-R22  .......T..SYEI.............L.G..S.G----SS...............N...A.........D.......TR..-------LLG-------------------..........  [125]
Pig12-VDJ-F37  .......T..SYEIF............L.G.YG.G----SS...G...........N...A.................R..-------YSGGAITR--LY-------TMN...........  [125]
PIG14-VDJ-R11  .......T..SYEI.............L.G.VSTG----S.....E..R.......N...A.............H....RIA-------IPMVLAIPPYY--------TM............  [125]
PIG14-VDJ-R17  .......T..SYEI.............L.G.VSTG----S.....E..R.......N...A.............H....RIA-------IPMVLAIPPYY--------TM............  [125]
PIG14-VDJ-R6   .......T..SYEI.............L.G.VSTG----S.....E..R.......N...A.............H....RIA-------IPMVLAIPPYY--------TM............  [125]
Pig3-VDJ-R6    .......T..SYEI.............L.G.YW.G----S................N...A...............W..R----------PLS.QIPTYYW-------AMN...........  [125]
Pig6-VDJ-F19   .......T..SYEI.............L.G.YS.G----SS...............N...A.................RIV-------FFYVVTY-GVID-------AMD...........  [125]
Pig12-VDJ-F9   .......T..SYEI.............L.G.WS.G----DS...............N...A................IR-------------------P-------ARES..........  [125]
Pig3-VDJ-R20   .......T..GYEI.............LSS.YR.G----ST...............N...A.................RFA-------D-------WTD---------MD...........  [125]
Pig6-VDJ-F36   .......T..SYEI.............L.GMYS.G----SGI..............N...A.................RYH-------TSPEL.IVYYD---------MD...........  [125]
Pig13-VDJ-R11  .......T.GSYEK.............L.G.YS.G----SN.H...........S.N...A.................R.--------VAIGMV.VAGRVRVS---.AMD...........  [125]
```

```
Pig13-VDJ-F2   .......T..SYEI............L.N..S.G----SS.....F.....M.S.N...A.......F.........R.R-------IGISMVIVDGR---------MD..........- [125]
PIG14-VDJ-R34  .......T..SYEI.......E.....L.R.YD.G----SV..........A....N...A.......N.........R.S-------IAI.MVLVAIEP------.AMD........... [125]
Pig13-VDJ-F27  .......S.NSYEIN...........L.G.YS.G----SS...............N...A.................R.R-------IAI.MVLVTWDM-------TGD........... [125]
PIG14-VDJ-F6   .......T..SYEI............L.G.YS.G----SS...............N...A............H....RKS-------LWW.MVLATQDP-------.MD........... [125]
Pig3-VDJ-R13   .......T..SYEI..........P..L.G.YSGG----SS.D.........V...N...A............H...VR--------------LYYRSRVNRY---.T.D........... [125]
Pig12-VDJ-F4   .......T..SYDIH...........L.N.YS.G----RS.D.............N.R..A.......L........VR--------------GNLG-----------.D........... [125]
Pig6-VDJ-R2    .......T..GYEIK...........L.G.YG.G----SILD.....E........N.ES.A.................R-------------CYSN---YYG------MD........... [125]
Pig12-VDJ-R6   .......S..SYEIT...........L.GLYM.G----SHF..P..A.....V.G.KRE..A....D.............R.----------FLCVRNDGNCYGG---DAMD........... [125]
Pig6-VDJ-F18   .......T..SYEIN.......R....L.G.Y..A----DIAD.P......V.....N.R..A.........G........S-------------E.RHG--LYA------MD........... [125]
Pig12-VDJ-F25  .......T..SYEIN...........L.G.Y..G----S..H.........V...N...A.................R.----------RGAM.VVVQFYA------MD........... [125]
Pig6-VDJ-R13   .......T..SYEI............L.GVDSG--------S.........T....N...A....T.............GRQALDKISMVLTIMITRGRGGPEY---.AMD........... [125]
Pig12-VDJ-F30  .......T..SYEI............L.STYSGG----KIAT.....R........N...Y...I..............-----------------RGR---VE---DLMN........... [125]
Pig13-VDJ-R12  ..A....T..SYDI............L.G.YSG.----SS.N.............N...A.................R.----EVDMVL.VMMTRPP---YY---.AMD........... [125]
Pig3-VDJ-R26   .......T..SYEI.....T....R..L.G.WS.G----.L..............N...A...V..............SC---------YRSSCYTYEGNWYP---GGMD........... [125]
Pig3-VDJ-R8    .......T..SYEI............L.G.YS..----SV...............N...A............H....SC---------YRSA.YH-----------GMD........... [125]
Pig6-VDJ-R42   .......T..SYSVN...........L.G.YSGA----SS.F.............N...A..................GCY------STYGTTCS-------Y---FG.D........... [125]
Pig6-VDJ-F23   .......T..SYEI.-..........LTC.YS.G----SS...............N...A................W.G---------NYG.TCSY------Y---.AMD........... [125]
Pig6-VDJ-F2    .......T..STLIT...........L.I.YS.T----SI...............N...A..................-----------------------SW---W..D........... [125]
Pig13-VDJ-F18  .......T..SYEIT.....Q.....L.G.YS.G----SS...............N...A.................R.K------SIAITMVLV-----SY---NA.D........... [125]
Pig3-VDJ-R30   .......T..SYEI............L.Y.TSNG----.S...............N...A............H....R----------GF---------FVY---SAVD........... [125]
Pig3-VDJ-R16   .......T..GYEI............L.G.LS.G----DN...............N...A............H....RV---------GYSYGASYYDPFQY---NAMD........... [125]
Pig3-VDJ-F20   ......VT..ITWIN...........Q.EFTS.G----SS...............N...A.......S....H....R----------GTDS------WVYY---.AMD........... [125]
Pig3-VDJ-F21   ......IT..SY.V............L.S.YS.G----SS............P..N...A.......S....H....R----------GADS------WVYY---.AMD........... [125]
Pig12-VDJ-F14  .......T..IYEIN...........L.G..S.G----SS...............N...A....G............RN--------YGIVWY----------------H........... [125]
PIG14-VDJ-F17  .......T..SYEIN...........L.Y.TATG----SS.D.............N...A....T......H...SRDT-------YGASYYG----DPYY---.SMN........... [125]
Pig3-VDJ-R34   .......T..VYPIN...........L.G.YG.H----SS...............N...A...D............GTLS------------LWGAECYYLA---SQMD........... [125]
Pig12-VDJ-F8   .....E.T..VTDIK.......Q....L...SS..----SS.V.............N...AH...............---V------------AYGDN---LD---G--D........... [125]
Pig12-VDJ-F17  .......T..SYPIG...........L.TN.N.G----SF...G.................A............PRAVELLA------------VAILATIFS-----VMD........... [125]
PIG14-VDJ-R40  .......T..SYPIG...........L.GV.N.G----SS...I...........N...A.......A.........R.C------------GGCWTSYVSG---DGMD........... [125]
PIG14-VDJ-F3   .......T..SYPIG...........L.D.SN.G----ST...T..A.........N...A.................RDV------------DYTYGVSYY-----TMN.....A...... [125]
PIG14-VDJ-R14  .......T..SDPIG...........L.R.FSGV----SS...........V....N...A............H....A---------------------YYE---DTM............ [125]
PIG14-VDJ-F2   .......T..SDPIG...........L.R.FSGV----SS...........V....N...A............H....A---------------------YYE---DTM............ [125]
PIG14-VDJ-F16  .......T..SDPIG...........L.R.FSGV----SS...........V....N...A............H....A---------------------YYE---DTM............ [125]
PIG14-VDJ-R24  .......T..GYPIG...........L.G.S..V----TT......A...L.....N..T.A....T.............GCT------------Y--CAGWYWE---FDMD........... [125]
Pig6-VDJ-R20   .......T.TVTYMN....T.......L.G.YSAG----.S............P.S.N...A.................R.---------YND--LHG-----------MN........... [125]
Pig6-VDJ-F22   .......TV.YRSMT.........M..L.C.YS.G----.-...T........F...N...A......P..........T.---------NGDDCYTGT----------MN........... [125]
```

```
Pig3-VDJ-R19   .......T..NYGIA............L.G.YSGG----TT...........V...N...A..................RDCY------RYGGTCYDDQA------INVMD............ [125]
Pig12-VDJ-F6   .......T..SSD......P.......L.R.YS.G----S-...V.......V...N...A..................RT---------------------------LSD............ [125]
Pig13-VDJ-R40  .......T..SYCM.............L.C.YS.G----SS...............N...A..................TP-----------G.CYSGYVGGEARY..AMD............ [125]
Pig13-VDJ-F6   .......T..SYGM.............L.C.YS.G----SS.............E.N...A..................T------------G.CYSG--------..VMD............ [125]
Pig13-VDJ-R35  .......T..NYNM.............L.Y.YS.G----SS...............N...A..................T.---------PYSDYYSGG-------EGAMD............ [125]
Pig13-VDJ-F17  .......T..SYSM.............L.C.YS.G----SS...............N...A....S.............T.---------RGT.CFSGG--------GFSD............ [125]
Pig12-VDJ-R16  ......LT..SYVM.............L.W.YS.G----SS...............N...A........S.........T.---------HGTSYDDG----------A.............. [125]
Pig13-VDJ-F5   ......YT..SYGM....R........L.G.YS.G----SS.............S.N...A..................R.---------E.SLY-------------AMD............ [125]
Pig13-VDJ-F24  ......YT..SYGM.............L.C.YS.G----SS...............N...A..................T.---------EGYLYYAAGY-----VAPQMD............ [125]
Pig6-VDJ-R14   .......T..SYEI.............L.G.YS.G----SS...............N...A..................RVTI------FAAIAVPTT---------LAMD............ [125]
Pig13-VDJ-F11  .......T..SYEI.............L.GMDS.G----SS...............N...A..................REII------VVNIAVP-----------.AMN.....I...... [125]
Pig13-VDJ-F16  .......T..SYEI.............L.G.YS.G----SS...............N...A..................R.SL------EVAEAVTVYSGGST----.AMD............ [125]
PIG14-VDJ-R27  .......T..SYEIK............L.G.YS.G----.S..............GN...A..................R.NI------SVAIAVGQIT--------RHMD............ [125]
Pig13-VDJ-R15  .......T..SYEI.............L.G.SAGG----DS.............K.....A..................RDIL------AVAIAAT-----------HAMD............ [125]
Pig13-VDJ-R14  .......T..SYEIN............L.G.YS.G----SS...............N...A...T..............TRTI------AVAMAIQIYPY--------AMD............ [125]
Pig13-VDJ-F13  .......T..TVTMN............L.C.YS..----SS...............N...A..................TESI------AAAIAVTSSP----------MD............ [125]
Pig13-VDJ-R16  ......YT..SYGIG...........SL.G.YSGG-----S...T.........K.N...A.................T------------TIPIMLVA----------MD............ [125]
Pig13-VDJ-F22  ......YT..SYGIG............T.G.YSGG-----S.............K.R...A..................RII---------AFPIMVTR----------MD............ [125]
Pig13-VDJ-F20  ......YT.RSYGIG............L.G.YSDG-----S...............N...A.....G............TFAP------DIFLKIGVTP----------KD............ [125]
Pig3-VDJ-R27   .......T..SWSVD............L.G.YS.G----TS..V............N...A...........R......R----DSSGCDS------------RPYFDVMY...........- [125]
Pig12-VDJ-F26  .......T..SSYIH............L...YSGG----SSPGH.......L........A..................R----KGAG---------------------.YV........... [125]
Pig12-VDJ-F35  .......T..STYIN............L.G.YD.G----SS.D.........V.S.N...A...V..............R-----RWTTA------------------VWDV........... [125]
Pig12-VDJ-F13  .......T..STYIN............L.G.YNTG----SS...............N...A.....N............R-----NIAVAGLVTPL---PTTLGRV..SMD.....T...... [125]
Pig3-VDJ-F1    .......T..STYIN............L.G.YS.G----S..D.............N...A..................R-----NGLRR-------------------MEV........... [125]
Pig6-VDJ-F26   .......T..STYIN............L.G.R.TG----.D..............SY.K.A..................R-.S--GGSCGA------------FDYGPI-D....S....... [125]
Pig12-VDJ-R9   .......T.INTYIN............L.G.YNTG----SS......M........N...A..................R-CYSYGGSCSS------------DTY.AI-NI........... [125]
Pig12-VDJ-F40  .......T..STYIH............L.G.HS.G----SG...............N...AF.......S.........R.CYIYGGSCWS------------NEHDQIMD............ [125]
Pig3-VDJ-F28   .......TLKSIYIN............L.SFPP.G-----S.............S.N...A..................RN------------------------IG.GMNV........... [125]
Pig13-VDJ-R21  .......T.NSYSII............L.G.YS.G----RS.............S.N...AS.L.....N.........RDCYSYGGSCPY--------------VG.GMD............ [125]
Pig3-VDJ-R28   .......T.TTTYIN............L...SGNG----.V...............N..SAA......T...A.H....R.VD------------------ISLVVG-SRTI........... [125]
Pig3-VDJ-R10   .......T..STYIN....L.......L..VSG.G----KD...............N...A.................VR..S------------------ITVVLAITSNI........... [125]
Pig6-VDJ-R3    .......T.NKTNIN............L.H.SS.G----SV...........N...N...A...............F..R-LG------------------AMVVVG---NR........... [125]
Pig3-VDJ-F2    .......T...GHIT............L.S.SRTG----SKII.........V...N..T.A..................R--------------------------G---TA........... [125]
Pig3-VDJ-R12   .......T..T.YIK............L.SVS.GG----ST...............N...A...................DS-------------------RGWSFTST--V........... [125]
Pig3-VDJ-F9    .......T..T.YIK............L.SVS.GG----ST...............N...A..................TNCWW-----------------SGGSCNDAGL............ [125]
Pig12-VDJ-F19  .......T..TTWIK............L...S..G----H..D.............N...A..................SDP-----------------------------R........... [125]
```

```
PIG14-VDJ-R10 T......T...TYI..A..........L.T.S.A.----AS...........V...N...A.................RRD------------------SGCANS-YVD-..........  [125]
PIG14-VDJ-F19 T......T...TYI..A..........L.T.S.A.----AS...........V...N...A.................RRD------------------SGCANS-YVD-..........  [125]
Pig3-VDJ-F5   .......TY..TYIH.I.........L...S..G----SN...V...........N...A...L............GRSQ------------------YRHYDSGCVD...........  [125]
Pig3-VDJ-R37  .......T..STYIN...........L...SS.G----.S...............N...A.................R-YY--SACYLG-----------------YSI..........  [125]
Pig13-VDJ-F23 .......T..STYIN...........L...S..G----.S...............N...AH................T.FFRCSGCYSDP----------------WD...........  [125]
Pig13-VDJ-R10 .......T..STYIN...G.......L.F...GG----AI.S.............N...A..............F..R.-----GCYIGY------------NC.DM............  [125]
Pig13-VDJ-F12 .......T..STYIN...G.......L.F...GG----AI.RH............N...A..............F..R.-----GCYVGY------------NC.DM............  [125]
PIG14-VDJ-F4  .......T..STGIN...........L..MS..G----DS.N.................A.................A.YS---GCYSGYVTPIPTSLTLLCSN..TMD...........  [125]
Pig3-VDJ-R39  .......T..TTYIN...........L.G.TG.G----.T...............N...A.................R.-ECHG----------------------ME....DFWGP..-  [125]
Pig6-VDJ-R4   ......IT..TTYIN...........L.G.DS.G----VS...............N...A...T..........C..GCLHCVGDDFVCKV---------------CMD..........-  [125]
Pig6-VDJ-F14  .......T..NTYIN...........L...S..G----.S.............K.N...A.M...............T.RDCYGGVWYGSC---------------AVD...........  [125]
Pig12-VDJ-R12 .......T..STYIN...........L...TA.G----.S...............N............I.........S.YD--AGRWYYS---------------AMD...........  [125]
Pig6-VDJ-F24  ..........STYIN...S.......L.GLF..G----.S...............N...A.......S.........R.RD---GCGYSR----------------GMD...........  [125]
Pig3-VDJ-F12  .......T..SSYIN...........L...S..G----.S...T...E.....K.N...A.................TDTTE--AV.IA.S------------VVGAMD...........  [125]
Pig6-VDJ-R28  .......T..STYIN...........L.G.W.GG----.S.H.T...E.....K.N...A....S............RTV--------------------------AMD...........  [125]
Pig6-VDJ-R35  .......T..STYIN...........L...S..G----.S...T...E.....K.N...A.................TCRSG--CYSGY.P-----------KSP.AMD...........  [125]
Pig6-VDJ-F3   .....V.T.TSTYIN...........L.R.S.G.----.G...T...E.....K.N...A.........G........R.CSG--CYVGYR--------------..SMD...........  [125]
Pig3-VDJ-R1   ..S....T..STYI............L...S..G----.S.......E.....K.K...A.................I.S-----IYGAR---------------.IMD...........  [125]
Pig6-VDJ-R30  .......TL.RTYI............L.TMS..G----.S...L.......T........A.................T.QG.--VVPIV.M.----------GVS.VPD...........  [125]
Pig6-VDJ-F34  .......T..STYIN...........L.RLS..G----.S.D.E...........N...A.................T.LG.--AVTVP-------------GWSV.MD...........  [125]
Pig6-VDJ-R32  .......T..STYIN...........L...S..G----.S.......E............A.................T.QRL--HI.V.-----------------MD...........  [125]
Pig6-VDJ-R29  .......T..STYIN...........L...S..G----.S...............N...A.................R.SCY--GDRCYFR-----------DD..AMD..........-  [125]
PIG14-VDJ-R2  .......T..STYIN...........L...S..G----.S...............N...A.................R--------RQL..-----------TP..AMD...........  [125]
PIG14-VDJ-F13 .......T..STYIN...P.......L...S..G----.S...............N...A................VR.-------TTRLR-----------KRQ.AMD...........  [125]
PIG14-VDJ-R39 .......T..STSIN......R....L.S.S..G----.S...............N...A............HF..R.NTW.--LV.TMTN-----------PHVLSMD...........  [125]
PIG14-VDJ-F8  .......T..STYIN...........L.H.S..G----.S....E.........S.N...A.................REFSM--LV.-------------------MD....A......  [125]
Pig6-VDJ-R11  .......T..STYIN...........L...S..A----DT...........V.S.N...A.................-------SG----------------LLW.AMDV..........  [125]
Pig6-VDJ-F27  .......T.RRTYIY...........L...S..G----SS..............S.N...A..................N-----SGGGSCYL----------CSN.AMD...........  [125]
Pig6-VDJ-R27  .......T..GSYIN...........L.T.SS.G----.SS......G....G.I.N...A..............H..T.-------LSM.LV----------AWG-AMD...........  [125]
Pig6-VDJ-F28  .......T..GSYIN...........L.T.SS.G----.SS......G....G.I.N...A..............H..T.-------LSM.LV----------AWG-AMD...........  [125]
Pig6-VDJ-F40  ......LT..KTYIN...........L.T.SI.G----.S.............K.N...A.M...............T.PCYKYDASCPAVV----------TYS.AMNF..........  [125]
Pig3-VDJ-F25  .......SI.STWIN...........L...TSGG----.V...............N...SAS................R.-----------------------------MDV..........  [125]
Pig6-VDJ-R5   .......T..STYIN...........L...S.DG----.G.............N.N...AS....R............R.VIERTMVLHAMMT-----SAEATTAQTAMD...........  [125]
Pig3-VDJ-F26  .......T.TSTYIN...........L..VSGAG----TN.......E.....K.S...A........G.........R.LLQIL----------------G---GL..D...........  [125]
PIG14-VDJ-R36 .......T.NSTYIN...........L...SD.G----.N.......RR.V..........A....S..........H..R----NL----------------G---VSSMN...........  [125]
Pig3-VDJ-F29  ......LT..STYIN...........L...S..G----.S...T..ME........N...A............H....RDFGYTY----------------GTEYQTVPN...........  [125]
```

```
Pig6-VDJ-R9     .......T..STYIN.I..........L.T.SL.G----.S...L...E..V.....N....A.............H....RADALR---------------------VIR.D...........  [125]
Pig12-VDJ-R7    .......T..RTDIN............L...SP.G----.NIH.....R........N....A..................RDIEP-------------------ILAVTR.D...........  [125]
Pig13-VDJ-F25   .......T..GAYIN............L...R.GG----.N.A.P...E........N....A..................RDIAP-------------------TIGVTLMD...........  [125]
PIG14-VDJ-R1    .......T.TRTYIN............L...SIGG----VI.H...........S.N....A....K.............RDDFSDY----------------CSASVCGME...........  [125]
PIG14-VDJ-R12   .......T..RHDIN............L...SIGG----VI.H...........S.N....A....K.............RDDFSDY----------------CSASVCGME...........  [125]
Pig13-VDJ-F10   .......T..STYIN............L.C.YS.G----.G.D....................A.............H....RP-----------------------IVDAMD...........  [125]
Pig12-VDJ-R5    .......S.N-------------------------------------..............R..A..................R-------WLLN---------------------..........  [125]
PIG14-VDJ-R18   .......T..NSDIN............L.TAT.DG----VS.W....................A..................R.QW-CYIWGV.C--------------SA--D...........  [125]
Pig6-VDJ-R39    .......T..STYIN............LTG.S..G----RS............G.........A..................TADLNCYEYGDSC--------------SDGMD...........  [125]
Pig3-VDJ-F19    .......T..SRHIN............L.G.S..G----SS..........I....N....A.................IN-----------------------F------V..........  [125]
Pig12-VDJ-R15   .......T.GSRHI.............L.GVR..G----.SP..........S....N....A..................G.AT.AVPATIYTWD.P-------RFRKTTMG...........  [125]
Pig12-VDJ-F23   .......T..NTWIN......E.....L.R.Y..G----SN............T..GN....A.......K..........A.QFDS--------------------ID.AM............  [125]
PIG14-VDJ-R21   .......T.RSTHIN............L..FYN.G----TN..........I....N....A........G..........A.YHDCRD-----------------IISVGVD...........  [125]
Pig3-VDJ-F22    .........STGIN.............L...N..G----SN...............N....A..E................R.-----------------------RLIVA.RV...........  [125]
Pig6-VDJ-R43    .......T..SYEIN............Q.S.VG.G----ST...V...........N....A..R.K..............G.LDNAVAI.LVP------------DRVRVM.V..........  [125]
PIG14-VDJ-R26   .......T..SYEIN............L.S.YS.G----SAA..............N....AT................KT.LSGAS-----------------------N...........  [125]
Pig6-VDJ-R31    .......T..TYPVI............L.Y.TR.G----.S...............N....A.V.................R.RA-----------------MVQFNILSAMD...........  [125]
Pig12-VDJ-R30   .......T..TYPIA............L...C-.G----.Y......................A..R...............R.-----------------------VVTSR--...........  [125]
Pig6-VDJ-R46    .......T..SY.V.............L.G.DSGS--.S.S......................A....T.............GVYS-------------GYSGVPSYYA---MD...........  [125]
Pig13-VDJ-R24   .......T..SY.V.............L.G.DSGS--.S.S......................A....TT............GFY--------------GTTSWSSFYA---TD...........  [125]
Pig6-VDJ-F1     .......T..SY.V.............L.G.DSGS--.S.S......................A....T.............G.I---------------AVAPFAIYA---MD...........  [125]
Pig3-VDJ-R35    .......T..SY.V.............L.R.DSGS--.S.S.V........L...........A.................VGDY------------------WEYASS---MD...........  [125]
Pig6-VDJ-R19    .......T..SYKV.............L.G.DSGS--.I.S......................A....T............TGCVS-------------SRSGAGCYDD.--MD...........  [125]
Pig6-VDJ-R25    ...A..IT..SW.M.............L.G.SSGS--.S.S..........L...........A....T.............GCLG-------------CYSGGGGYFR---MD..........-  [125]
Pig12-VDJ-R3    ...A.R.A..SYGVV............L.G.R-IS--IS.N....A...............S.A....T.............GCA--------------EYYF-PYYYS---VD...........  [125]
Pig12-VDJ-R19   .......G..SYGVV.I..........L.G.R-IS--SS.N....E..........A..RS.A....T.............GCN--------------EYWF-PYYYS---ID...........  [125]
Pig12-VDJ-F3    .......G.RSVGVV............L...R-IS--IS.N..................R..A....A.............GCA--------------EYYF-PYYYS---MD...........  [125]
Pig12-VDJ-F5    .......G.RSVGVV............L...R-IS--IS.N..................R..A....A.............GCA--------------EYYF-PYYYS---MD...........  [125]
Pig12-VDJ-R10   ......IT..SY.V.............L.G.DSGS--.S.S......................A....T.............GCA--------------EYWF-PYYYS---MD...........  [125]
Pig12-VDJ-R28   ......IT..SY.V.............L.G.DSGS--.S.S......................A....T.............GCA--------------EYWF-PYYYS---MD...........  [125]
Pig6-VDJ-F16    ......IT..SD.V.............L.S.DSDY--.SDS......................A....T......S.H....GCE--------------DYNVGPSCYL---AD...........  [125]
Pig6-VDJ-F9     ......IT..SY.L.............L.G.DSGTYI.TDS......................A........I.........G.CS-------------DSYGASCYDD..AMD...........  [125]
Pig3-VDJ-R23    .......TLRSF.LI............L.G.D.GS--.R.SD.C...................A...................SYS-------------GWYS--GYWA---ME...........  [125]
Pig3-VDJ-R38    .......T..SYDII............L.G.DSGV--.T.I......................A...........R......GCEE-------------CYNGFFVFGYS--MD...........  [125]
PIG14-VDJ-F11   ..........RYEVT............L.G.DD----GT.T.V.E...R..............A..E..N..S.........MS----------------YTYGISYDYCG-MDR..........  [125]
PIG14-VDJ-F14   .......T..RYEVT............L.G.DD----GT.T.V.....R..............A..................M.----------------YSYGISYDYCG-MDR..........  [125]
```

```
Pig12-VDJ-R2   .......T..SYEI............L.G.S..GR-GT.S....................A.................VFRD-------------CYIYGITC-YGA-MD...........  [125]
Pig12-VDJ-R23  ...D...T..SYDM............L.W.D.----.GSG...............N...A.................T.-----------------TNRYGYYP--VMDR..........  [125]
PIG14-VDJ-F1   ......ITV.SYPIG.......N....L.DTS..G----.S.............K.N...AT................T.-----------------LMVLSSRT.GAMD...........  [125]
PIG14-VDJ-F5   ......ITV.SYPIG.......N....L.DTS..G----.S.............K.N...AT................T.-----------------LMVLSSRT.GAMD...........  [125]
Pig6-VDJ-F31   .......T..SYEIT...........L.D.S..G----.Y...............N...A.................T.-----------------YIHR------KMDF..........  [125]
Pig3-VDJ-R14   ......YT..SYPIY...........L.S.S.AG----AS...........G.I.N...A..........A.....R.-----------------DAYG----VRSMD...........  [125]
Pig3-VDJ-R18   .......T..NYEI............L...S.IS----.SI..T...E.....K.N.L..A.................  -----------------IAMVRVAFG..GMD...........  [125]
Pig13-VDJ-F4   .......T..SYEI............L...S..G----.S...............N...A...V.............R.KI-------------AIAMVLVAKTH.-MD...........  [125]
Pig6-VDJ-R44   .......T..SYEI............L...S..G----SS.G..................A....I.......S...AT-----------------FVMVNSYLVLR.CNM..........  [125]
Pig12-VDJ-R21  .......TL.SYEI............L.N.I..G----DY.A.............N.H..A....G...........TD-----------------LWTIN--------...........  [125]
Pig3-VDJ-R33   .......T..SY.VN...........L.GMSV.A--KT--SQ............K.N...A....N........F.VR---------------------------GGMD...........  [125]
Pig3-VDJ-R36   .......T..SS.V............L.GVEGDS--.I................K.N...A.................VTE----W----------LVTMM----TWNAMD...........  [125]
Pig12-VDJ-R25  .........IW.V.............L.GKNG..--SS-TAC.L..........K.N...A......T..........RC----G----------TVT--------RRMD...........  [125]
Pig6-VDJ-F5    .......T..GY.VR...........L.G.DK.G--SS--..............K.N...A.................TR-------------------------HITRDG...........  [125]
Pig3-VDJ-R2    .......T..SY.V............L.E.Y..G--SS--..............K.N..K.A.................RD----C----------YRYDVTCTGTE.AMD...........  [125]
Pig6-VDJ-F21   ......LT..SY.V............L.G.YS.G--SS--..............K.N...A.................-------C----------YWF--------LYMD...........  [125]
Pig6-VDJ-R24   ......IT..TY.V............L.D.E.AG--.SSS..............K.....A.................  ------------------LQLRWWGRG.DPMD...........  [125]
Pig6-VDJ-F8    ......IT..SY.V............L...S..G----DS...............N...A.................A.RA--R----------AMVLHLWGN-..GMD...........  [125]
Pig6-VDJ-F6    .......T..SY.VT...........L.G.S..G----SV.W.G..........K.N...A.................R.----------------------GCG.CFMD...........  [125]
Pig3-VDJ-R11   .......T..SF.VN...........L.R.TS.G--GV--.E.............N...A....G.T.........TR--------------------GWY------ISF..........  [125]
Pig3-VDJ-F30   ......IT..SD.VN...........L.G.SK.A--SV--VF.................GA.......T..........T.DCYRG----------GTSCGWDSAIAVGMTF..........  [125]
Pig12-VDJ-F24  .......T..SD.VT....P.......L.I.YS.G--SS--....................A.......K.......F.RT.------------------------------GY.........P-  [125]
PIG14-VDJ-R20  .......T..SY.V............L.G.DN.G--SS--....................A..............H...T.RDYW------------SSGDNYLID..YMS...........  [125]
Pig3-VDJ-R3    .......T..SY.V............L.Y..S.----GRY.D.............EN...A.................R.LAYG----------------------AIMD...........  [125]
Pig3-VDJ-F18   .......T..SY.V............L.Y..S.----GRY.D.............EN...A.................R.LAYG----------------------AIMD...........  [125]
Pig6-VDJ-R17   .......T..SY.V............L.G.ES.----GSY...T..................A.................V.LNCGN--------------CYLWGH--.DSD...........  [125]
PIG14-VDJ-R23  .......T..RY.V............L...SS.----....D....................A.......N.........IENCLTW----------GALCYAWGG--.AID...........  [125]
Pig3-VDJ-F11   .......T.TRY.V............LGS.S.G----GIA...............N...A...................YIG--------------CYLFG----RVD...........  [125]
Pig13-VDJ-R31  .......T...Y.V............L.N.DSDI--.S.D...............K.S...A.................-.QSSG--------------CYIGYGCQ.ATD...........  [125]
PIG14-VDJ-R35  .......T..SYRVR...........L.C.HS-----S.T..H.............E...A...K..K..........V.QGGG--------------CYSG-----AMD...........  [125]
Pig3-VDJ-F10   ..........Y...............L.S.S..G--SS--................T...A.................R...TP---------------VLVTKY..GMD...........  [125]
Pig12-VDJ-F20  ..........Y...............L.Y.TS.G--SS--................N...A.................T.--------------------------VGIGA..........  [125]
Pig12-VDJ-R13  .........GYP..............L.T.SI.G--SN--................N..M..................RDAG--------------------HGCGNTCRS..........  [125]
Pig6-VDJ-F33   .......T..SYVV............L.G.S.TG--FT--.......R......K.N...A........S.E.......R.-WG------------------CPNYRASFDA..........  [125]
Pig6-VDJ-F37   .......T..SYKL............L...S..G--TS--..............K.N...A.................R.SW.------------------SYYD..T.DF..........  [125]
Pig12-VDJ-F27  .......T..NKVV............L.G.S..G--SH--................N...A.................TT----------------------IAVKRGVDF..........  [125]
```

Pig3-VDJ-R25 .......T..SYNMI............LIR.SSGG----SS..............K.N...A..................R.NS.Y----------------------.AMD........... [125]
Pig12-VDJ-F38 .......T..SY.LN............L...SGGG----RS...V...N........N...A..................T.HS.DGL------------SCGGWSYN.IAD........... [125]
Pig13-VDJ-F29 ..........SYNMI............L.L..NNN----DS...........VL..N.H..AW...........H....R-----------------------HGWH..D........... [125]
PIG14-VDJ-F20 .......T..SYGIG............L...SN.G----.S................N........L..............R.DVGC------------------YSGVCA.I........... [125]
Pig3-VDJ-F3 ...A...T..SYNVI............L...DY.SY--T.S.S..............N...A..............V..VIDF----------------------------.C......... [125]
Pig3-VDJ-F27 ...A...T..SYNVI............L...DY.SY--T.S.S..............N...A..................T.YSRSY---------------FDGWYM.SMD........... [125]
Pig6-VDJ-F29 .......T..SYDL.............L.T.S.TG----.S.SH.........V...N...AF.................TKTYSS------------------GWWFSAI............ [125]
Pig12-VDJ-R29 .......T..SYQI.............L.CFSSVT----.N.F..............N...A........A.........T.RLSS-----------------SCGIDRVIN........... [125]
Pig12-VDJ-R4 .......T..SYRMR............L.S.SSG-----.SSD..............N...A.................TSSVS.G----------------GS-----PIK........... [125]
Pig12-VDJ-R22 .......T..SYRMI............L.C.TS.S----.S.D...L.........N...A.................TT.LGTG----------------CYDLIH.AMDI.......... [125]
Pig3-VDJ-F13 .......T..STGII............L.EVTEDG---GL-...V.......VV.K.N...A....D...........T-------------------------LYLTY.D........... [125]
Pig3-VDJ-F23 .......T..STGII............L.EVTEDG---GL-...V..A....VV.K.N...A....D...........T-------------------------LYLTY.D........... [125]
Pig6-VDJ-R18 .......A..SY.V.............L.EVTE.G---GIS.F............KSN...A....N..I.........R---GC------------------GSC.SGFV........... [125]
Pig12-VDJ-F10 .......T..SY.VN............L.G.SAGG---A.-................N...A..HVT............LRRDIAY------------------QDYLHGK-........... [125]
PIG14-VDJ-R25 .......T..STYAD.......M....L...EP.G---GI-...........F...N...A..H..............G------------------------TDLLTPKN........... [125]
Pig3-VDJ-F16 .......T.RSKPIG............L.D.D-.G----.S..V.........V...N...A.......K..........SRLLWGG----------------VSMPQ.AMD........... [125]
Pig13-VDJ-F26 .......T..SYEI.............Q.S.DI.G----SS................N...A..................IRIVGN--------------------YH.AMD........... [125]
Pig6-VDJ-R37 .......T.RTTEM.............L.G.WS----SSSSLS..E...........N..M.A.................-----------------------IPLAAN.A.Y........... [125]
Pig6-VDJ-F32 .......T.TT.ETH...........SLTF..SGT--SSSS...............S..........................RCYT------------------YGIGCN.A.D........... [125]
Pig12-VDJ-R20 ...........................L.Y..SGS--.S.S................N...A..................ILKR------------------LWVAYP--Q........... [125]
Pig13-VDJ-R30 .......T..SYVL.............L.G.HSGS--.T.S.......................A..................G.KRKC-----------YRYDPTWVDCLSAMD........... [125]
Pig13-VDJ-F9 ......IT..RY.V............SL.F..SGS--.VDS.N.T..................S.......S.........R.LWYS-----------------RDSSLG.RMD........... [125]
Pig6-VDJ-F7 ..........SYGVG...........SL.SR.SGS--.I.S...............S..........................R.LYS---------------AVAKGGG---LMD........... [125]
Pig6-VDJ-F11 ..........SYGVG...........SL.S..SGS--.I.S...............S.........................SRV.WS---------------FFS-------AMN........... [125]
Pig12-VDJ-R24 .........RSYGVG...........SL.S..SGS--.L.S...............S.N...A..................RAALP--------------PVLVGVES..PMD........... [125]
Pig6-VDJ-R22 ..........SYGVG.......R...SMEC..N.S--.T.S...L..E.......S.......E.....R.A.....HQS.VWCKLP-------------------LPAAMD........... [125]
